# Genomic attributes of homology-directed DNA repair deficiency in metastatic prostate cancer

**DOI:** 10.1172/jci.insight.152789

**Published:** 2021-12-08

**Authors:** Navonil De Sarkar, Sayan Dasgupta, Payel Chatterjee, Ilsa Coleman, Gavin Ha, Lisa S. Ang, Emily A. Kohlbrenner, Sander B. Frank, Talina A. Nunez, Stephen J. Salipante, Eva Corey, Colm Morrissey, Eliezer Van Allen, Michael T. Schweizer, Michael C. Haffner, Radhika Patel, Brian Hanratty, Jared M. Lucas, Ruth F. Dumpit, Colin C. Pritchard, Robert B. Montgomery, Peter S. Nelson

**Affiliations:** 1 Divisions of Human Biology,; 2 Clinical Research,; 3 Vaccine and Infectious Disease, and; 4 Public Health Sciences, Fred Hutchinson Cancer Research Center, Seattle, Washington, USA.; 5 Department of Laboratory Medicine and Pathology and; 6 Department of Urology, University of Washington, Seattle, Washington, USA.; 7 Dana-Farber Cancer Institute, Boston, Massachusetts, USA.; 8 Division of Oncology, Department of Medicine, University of Washington, Seattle, Washington, USA.

**Keywords:** Oncology, DNA repair

## Abstract

Cancers with homology-directed DNA repair (HRR) deficiency exhibit high response rates to poly(ADP-ribose) polymerase inhibitors (PARPi) and platinum chemotherapy. Though mutations disrupting *BRCA1* and *BRCA2* associate with HRR deficiency (HRRd), patterns of genomic aberrations and mutation signatures may be more sensitive and specific indicators of compromised repair. Here, we evaluated whole-exome sequences from 418 metastatic prostate cancers (mPCs) and determined that one-fifth exhibited genomic characteristics of HRRd that included Catalogue Of Somatic Mutations In Cancer mutation signature 3. Notably, a substantial fraction of tumors with genomic features of HRRd lacked biallelic loss of a core HRR-associated gene, such as *BRCA2*. In this subset, HRRd associated with loss of chromodomain helicase DNA binding protein 1 but not with mutations in serine-protein kinase ATM, cyclin dependent kinase 12, or checkpoint kinase 2. HRRd genomic status was strongly correlated with responses to PARPi and platinum chemotherapy, a finding that supports evaluating biomarkers reflecting functional HRRd for treatment allocation.

## Introduction

Prostate cancer (PC) is a common age-associated malignancy that is now recognized to comprise distinct phenotypic and genomic subtypes (1–3). Deep molecular profiling of metastatic PC (mPC) has identified several subtypes categorized by defects in DNA repair processes, most commonly mutations in genes such as *BRCA2* involved in homology-directed DNA repair (HRR; refs. 1, 4). As with cancers of the breast, ovary, and pancreas, germline mutations in *BRCA1* and *BRCA2* associate with an increased risk of developing PC, and approximately 5% of men with mPC carry inherited pathogenic mutations in these genes (5). In addition, somatic alterations in DNA repair genes occur in approximately 20% of mPCs (6, 7). Preclinical studies of other genes recurrently mutated in mPC, such as chromodomain helicase DNA binding protein 1 (*CHD1*) and speckle type BTB/POZ protein (*SPOP*), indicate they may mediate HRR deficiency (HRRd; refs. 8–10).

Of clinical relevance, mPCs with HRRd are associated with exceptional responses to platinum chemotherapy and to inhibitors of poly(ADP-ribose) polymerase (PARP) 1 (11–13). However, there are subsets of patients with mutations in HRR genes that demonstrate primary resistance to these drugs and other patients without documented alterations in HRR genes who exhibit deep and prolonged responses (14–16). Consequently, identifying tumor features that accurately predict HRRd and consequent vulnerability to genotoxic drugs would enhance clinical care by improving treatment allocations.

Pan-cancer analyses have identified patterns of somatic mutations in cancer genomes, termed mutational signatures, that are associated with specific defects in DNA repair processes such as HRR or mismatch repair (MMR), environmental exposures to mutagens such as UV light or tobacco, and mutational patterns with unknown etiologies (17, 18). While mutational processes exert effects in benign cells, they are more readily evident in cancers because of clonal expansion. To date, more than 50 single base substitution (SBS) signatures have been identified. Other signatures comprise structural DNA alterations (18). In addition to providing insights into the mechanisms responsible for the accumulation of DNA sequence errors, mutational signatures have potential utility in tumor classification and may serve as biomarkers predictive of specific treatment responses. In this context, Catalogue Of Somatic Mutations In Cancer (COSMIC) mutational signature 3 (CSig3) is associated with HRRd caused by a variety of genomic and epigenomic alterations in well-characterized HRR pathway genes, including *BRCA1*, *BRCA2*, partner and localizer of BRCA2 (*PALB2*), and RAD51 paralog D (*RAD51D*) (17, 19, 20). However, CSig3 also occurs in tumors without alterations in known HRR genes, of which a subset respond to therapies exploiting HRRd (19–22).

In this study, we sought to assess the type and prevalence of mutational signatures in mPC. In view of potential clinical relevance as predictors of PARP inhibitor and platinum sensitivity, we focused on mutational signatures of HRRd and sought to identify germline and somatic alterations that could underlie their genesis and determine associations with treatment responses.

## Results

### Mutational signatures associated with aging and HRRd are common in mPCs.

**Figure 1:**
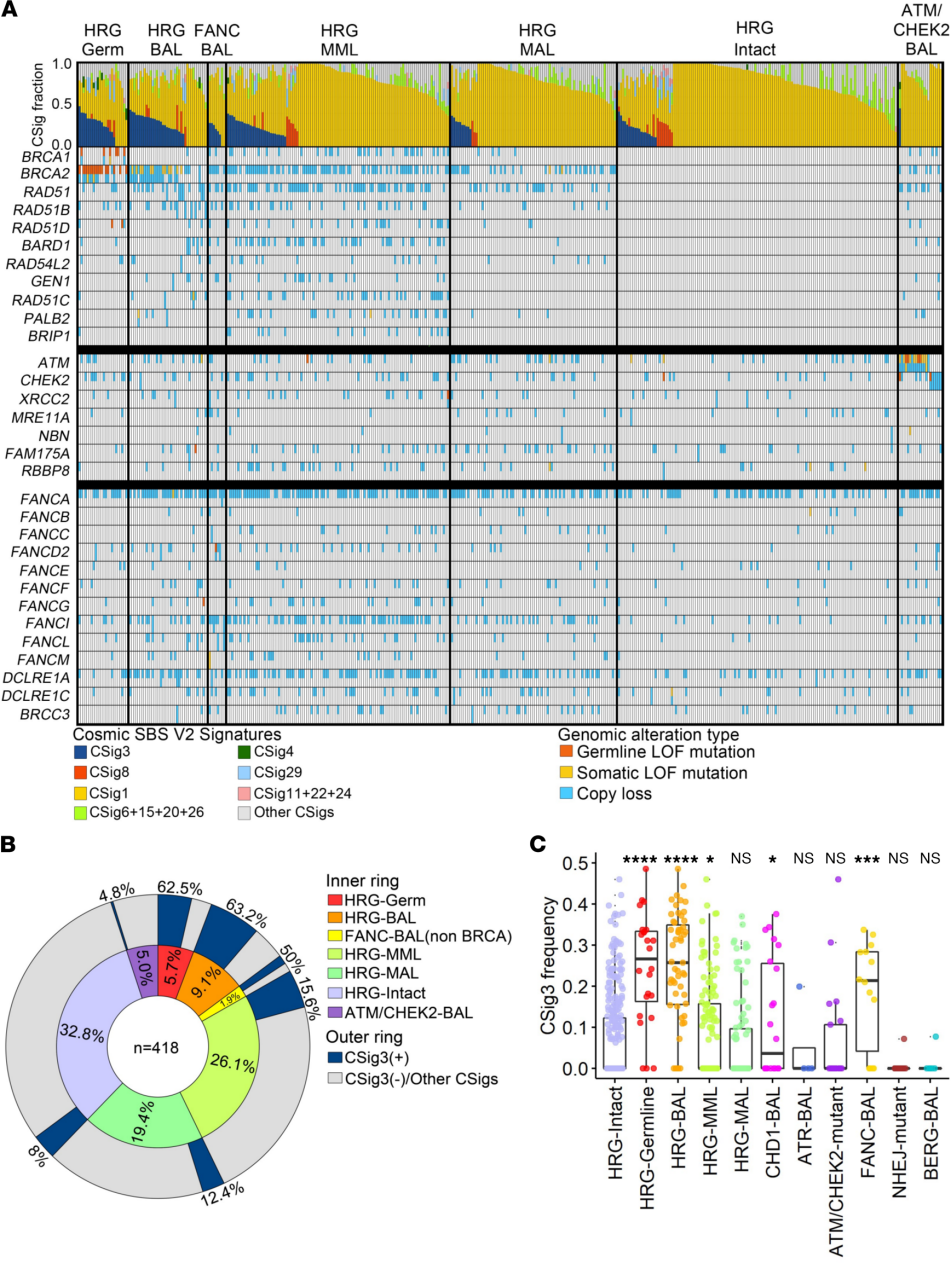
Metastatic prostate cancers have a high frequency of Catalogue Of Somatic Mutations In Cancer base substitution signature 3 associated with homology-directed DNA repair deficiency. (**A**) Distribution of COSMIC single base substitution mutational signatures (CSigs) across 418 mPCs. Tumors are grouped by homology-directed repair gene (HRG) germline mutation (HRG-Germ), HRG biallelic somatic loss (HRG-BAL), Fanconi anemia gene biallelic loss (FANC-BAL), HRG multiple monoallelic loss (HRG-MML), HRG monoallelic loss (HRG-MAL), no HRG aberrations (HRG-Intact), and tumors with a biallelic *ATM* or *CHEK2* loss (ATM/CHEK2). The classes of COSMIC mutation signatures are color-coded, and tumors are ordered in decreasing frequency of COSMIC signature 3 fraction (CSig3). (**B**) The distribution of CSig3 positivity and 7 genomic subclasses related to DNA repair in 418 metastatic prostate tumors. (**C**) Frequency of CSig3 in 418 metastatic PCs with alterations in genes involved in DNA repair. Comparisons of each alteration group with the no-HRG-aberration group by 1-sided Wilcoxon rank test with Benjamini-Hochberg multiple-testing correction shown on plot (FDR * ≤ 0.05; *** < 0.001; **** < 0.0001; NS > 0.05).

To assess the landscape of mutational processes present in mPC, we evaluated whole-exome sequences from 418 metastatic tumors for previously defined mutational patterns of SBSs termed COSMIC mutational signatures (CSigs; ref. 17). A subset of these CSigs, occurring in the context of 96 possible trinucleotide positions, have been assigned to specific mutagens or reflect defects in aspects of DNA repair mechanisms. We identified signatures associated with 5 mutational processes present in at least 5% of tumors (Figure 1A): signature 1 (CSig1), attributed to spontaneous deamination of 5-mC and associated with aging, present in 99% of tumors; signature 3 (CSig3), attributed to HRRd, present in 19.6% of tumors; unassigned signature 8 (CSig8), present in 7% of tumors (~2% of tumors had both CSig3 and CSig8); and signatures 6, 15, and 26, attributed to DNA MMR defects, present in 1.4%, 10.6%, and 1.9% of tumors, respectively. Signature 29, attributed to tobacco exposure, was identified in 6.5% of tumors. In addition, 2 signatures were present in a small number of tumors: signature 2 (2.4%) and signature 4 (1.2%). Notably, CSig patterns associated with mutational processes prominent in other tumor types, such as signature 13, associated with APOBEC mutagenesis found in breast and urothelial cancers, and signature 10, prominent in colorectal cancers, were either extremely rare or not present in mPCs.

### CSig3 is associated with genomic aberrations in HRR pathway genes in PCs.

Prior studies determined that combinations of mutation, copy loss, or epigenetic silencing resulting in biallelic loss of *BRCA1*, *BRCA2*, *PALB2*, or *RAD51C* in breast cancers associate with signatures of HRRd, predominantly CSig3 (19, 20). Overall, 19.6% (*n* = 82) of mPCs exhibited high CSig3 fractions exceeding 20% of the tumor single nucleotide variants (SNVs) with at least 50 mutations attributable to CSig3, hereafter designated CSig3(+) (Figure 1A). To determine an underlying cause for CSig3 activity, we evaluated each tumor for aberrations in 1 of 11 genes with well-described roles in mediating HRR: *BRCA1*, *BRCA2*, *RAD51*, *RAD51B*, *RAD51C*, *RAD51D*, *RAD54L2*, BRCA1 associated RING domain 1 (*BARD1*), GEN1 Holliday junction 5′ flap endonuclease (*GEN1*), *PALB2*, and BRCA1 interacting helicase 1 (*BRIP1*), hereafter designated as a core HRR gene (HRG; Figure 1, A and B). We excluded DNA damage sensors, serine-protein kinases ATM and ATR, and break site preprocessing MRE11/RAS50/NBS1 complex genes from this list and analyzed them separately (Supplemental Figure 1; supplemental material available online with this article; https://doi.org/10.1172/jci.insight.152789DS1). Overall, 45.1% (*n* = 37) of the CSig3(+) tumors had core HRG-BAL through mutation or copy loss: *BRCA1* (*n* = 2), *BRCA2* (*n* = 31), *RAD51* (*n* = 1), *RAD51B* (*n* = 0), *RAD51C* (*n* = 1), *RAD51D* (*n* = 0), *PALB2* (*n* = 2), *BARD1* (*n* = 0), *RAD54L2* (*n* = 0), and *GEN1* (*n* = 1; Figure 1, A and B). Of these, a germline mutation was present in 36% (*n* = 13). HRG-BAL tumors exhibited significantly higher CSig3 activity compared with tumors without these events (*P* < 0.0001; Figure 1C). Several CSig3(+) tumors had biallelic loss of other genes associated with HRR, but not designated as a core HRG, including the FA pathway genes *FANCC* (*n* = 1), *FANCD2* (*n* = 1), *FANCI* (*n* = 1), *FANCL* (*n* = 1), and *FANCM* (*n* = 1).

### PCs with HRR gene aberrations and CSig3 activity are associated with structural genomic alterations and increased mutation burden.

**Figure 2:**
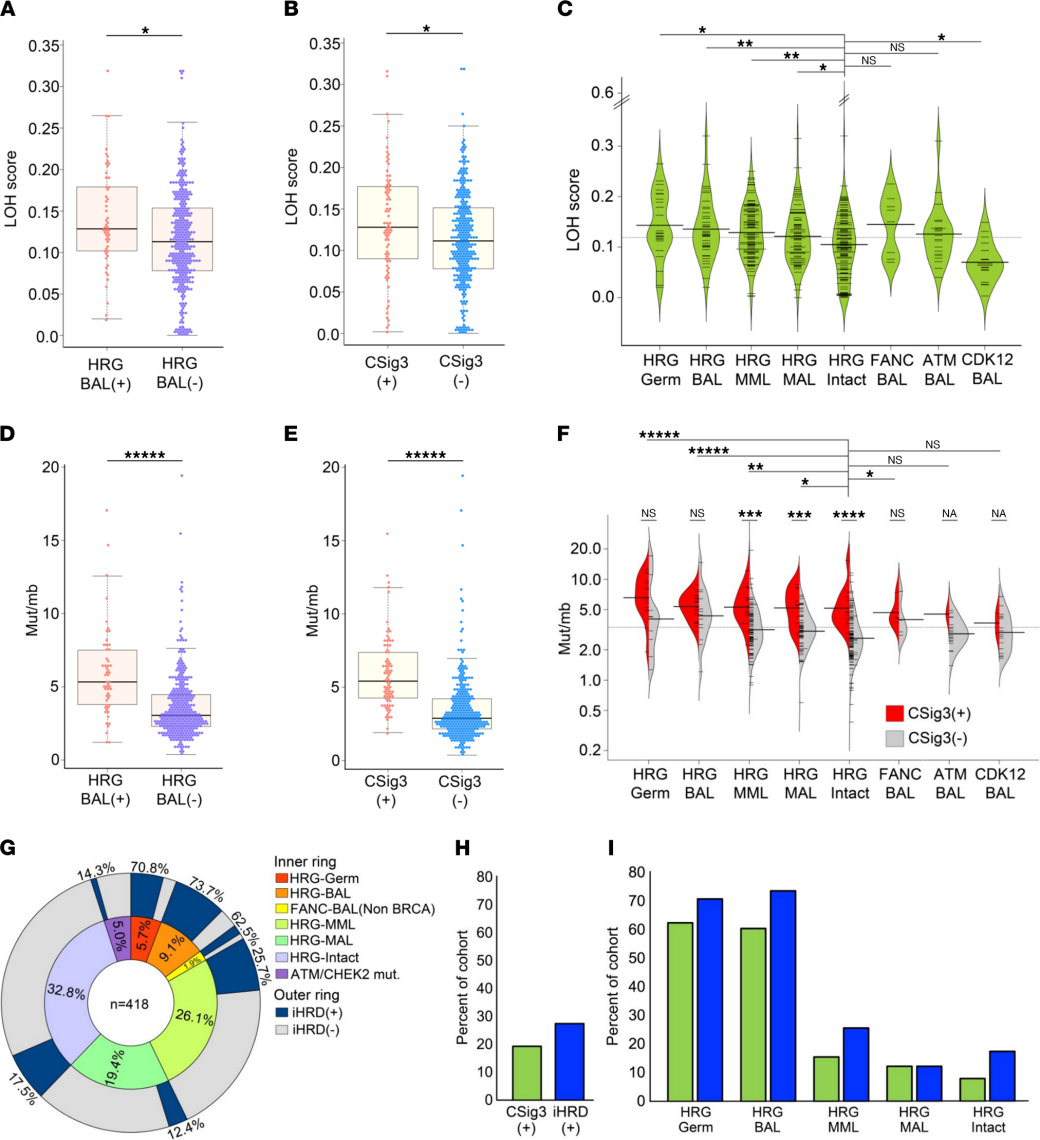
Loss of heterozygosity and somatic mutation burden associated with HRR deficiency mutation signatures. (**A**) Loss of heterozygosity (LOH) scores in tumors with HRG-BAL (*n* = 53) versus tumors without HRG aberrations, excluding those with hypermutation (*n* = 349) (*P* < 0.01). (**B**) LOH scores between tumors classified as CSig3(+) (*n* = 81) and CSig3(–) (*n* = 317) (*P* < 0.03). (**C**) LOH score distribution across tumors with molecular aberrations involving DNA repair processes. Compared with LOH scores of tumors without HRG alterations (HRG-Intact), LOH scores are significantly higher in tumors with HRG germline loss (HRG-Germ), *P* = 0.005; biallelic loss of a core HRG (HRG-BAL), *P* = 0.014; monoallelic loss of multiple core HRGs (HRG-MML), *P* = 0.0009; and biallelic ATM alteration, *P* = 0.043. Tumors with biallelic cyclin dependent kinase 12 (*CDK12*) loss have LOH scores significantly below tumors with other HRG aberrations (*P* = 0.029). (**D** and **E**) Somatic single nucleotide variants (sSNVs) in tumors (**D**) with HRG-BAL (*n* = 54) or (**E**) classified as CSig3(+) (*n* = 81) versus CSig3(–) (*n* = 317) (*P* < 0.00001). (**F**) Distribution of sSNVs across tumors with molecular aberrations involving DNA repair processes. Compared with sSNVs/Mb in tumors without HRG (HRG-Intact) (*n* = 3.3 ± 2.3), sSNVs/Mb are significantly higher in tumors classified as HRG-Germ, HRG-BAL, and HRG-MML (*P* < 0.01). Within HRG-MAL and HRG-Intact, tumors classified as CSig3(+) had greater numbers of sSNVs compared with CSig3(–) tumors (*P* < 0.001). (**G**) Stratification of mPCs by iHRD classification status across germline and somatic alterations in genes involved in DNA repair (*n* = 418). (**H** and **I**) Frequency of CSig3(+) and iHRD(+) tumors in (**H**) the entire cohort of 418 tumors and (**I**) each genomic subclass. (**A**–**F**) Mann-Whitney *U*. FDR: * ≤ 0.05; ** < 0.01, *** < 0.001, **** < 0.0001, ***** < 0.00001.

Prior studies have determined that defects in HRR are associated with genomic instability manifested by structural genomic alterations (23–25). Specifically, in breast and ovarian carcinomas, HRRd is associated with genomic patterns of loss of heterozygosity (LOH) encompassing regions greater than 15 Mb, but less than an entire chromosome (25). HRRd LOH assessments are associated with responses to platinum-based chemotherapy and PARP inhibitors (PARPi; refs. 25–28). To determine if genomic structural alterations associate with HRRd in metastatic castration-resistant prostate cancers (mCRPCs), we assessed the extent of genomic LOH in each tumor by calculating the ratio of exome sequence comprising LOH regions compared with total exome sequence, an approach that approximates the use of SNP array-based measurements to establish LOH scores (25). Overall, CSig3(+) tumors exhibited significantly higher LOH scores compared with CSig3(–) tumors (mean 0.133 ± 0.07 vs. 0.116 ± 0.06; *P* < 0.017; Figure 2, A and B). Further, LOH scores were higher in HRG-BAL tumors and those with HRG-MML compared with HRG-intact tumors (Figure 2, A and C, and Supplemental Tables 2–4). In agreement with recent reports, PCs with tumor protein p53 (*TP53*) loss also exhibited high LOH scores (mean = 0.134; Supplemental Table 3 and ref. 29), whereas those with biallelic *CDK12* loss had LOH scores significantly lower than tumors without HRG aberrations: 0.070 vs. 0.109, respectively (*P* = 0.014; Figure 2C).

In addition to a higher fraction of the genome involved in LOH events, CSig3(+) tumors were also notable for significantly greater numbers of somatic mutations, with a mean of 5.94 mut/Mb, compared with 3.47 mut/Mb in CSig3(–) tumors, after excluding hypermutated tumors (defined as ≥20 mut/Mb; *n* = 20) (*q* < 0.00008) (Figure 2, D–F). Hypermutated tumors were generally CSig3(–), with the single CSig3(+) tumor also notable for biallelic *RAD51C* loss. Tumors with biallelic *CDK12* loss did not exhibit higher numbers of mutations.

### Integrating HRRd-associated genomic parameters to identify tumors with functional HRRd.

Studies of ovarian and breast carcinoma indicate that no single genomic parameter accurately reflects HRRd or consistently predicts responses to therapeutics expected to be effective in the context of HRRd (27). Several features, including genome-wide measures of LOH, telomeric allele imbalance, large-scale state transitions, and mutation signatures, are each associated with *BRCA1/2* status, and combining multiple parameters has been shown to distinguish HRRd tumors from non-HRRd tumors (19, 21, 27). Structural genomic alterations are associated with *BRCA1/2* loss in PCs, suggesting that integrating such metrics may further improve mPC HRRd classification (29, 30).

We evaluated 6 genomic features acquired from the whole-exome sequencing (WES) data in order to develop an approach for classifying HRRd in mPC: the CSig3 score, CSig8 score, LOH score, total number of somatic mutations, tumor ploidy, and total number of genomic segments altered. As these features have variable scales of measurement and may not be linearly correlated, we used a nonlinear Gaussian kernel support vector machine model to develop an integrated assessment of HRRd (iHRD). We trained the iHRD classifier using 48 mPC tumors with biallelic loss of a core HRG against 190 mPC tumors that lacked any HRR gene aberrations, resulting in a misclassification rate of 2% (see Methods).

We applied the iHRD parameters to classify the Stand Up To Cancer (SU2C) mPC tumors: overall, 115 of 418 tumors (27.5%) were called iHRD(+) (Figure 2G and Supplemental Table 1). Of the 82 CSig3(+) tumors, 78 were iHRD(+) and 4 were iHRD(–). Conversely, of the 115 iHRD(+) mCRPCs 37 were CSig3(–) (Figure 2, H and I). Of 55 HRG-BAL tumors, 43 (78%) were iHRD(+): 15 of 17 tumors with HRG germline mutations and 28 of 38 with somatic HRG-BAL. Notably, 7 tumors with germline HRG mutations were classified as iHRD(–): 3 *BRCA1*, 2 *BRCA2*, and 2 *RAD51D*, and each of these tumors also were classified as CSig3(–).

### Metrics of HRRd are associated with CHD1 loss but not with alterations in ATM or CDK12.

We next sought to identify other genomic alterations that could underlie HRRd in mPC. Previous studies have implicated *CDK12*, *ATM*, *CHEK2*, *CHD1*, *SPOP*, and ribonuclease H2 subunit B (*RNASEH2B*) in promoting genome stability and regulating aspects of HRR (8–10, 31, 32). To determine associations between CSig3(+) and iHRD classification with an underlying genomic event, we identified a comparator group of 121 tumors without biallelic or monoallelic loss of any gene with established associations with HRRd, and excluded tumors with confounding events including hypermutation, x-ray repair cross complementing 2 loss, or non-homologous end joining (NHEJ) gene alterations: hereafter the HRR-Reference group. In this HRR-Reference group, 11 (9%) tumors were classified as CSig3(+), and 23 (19%) tumors were iHRD(+). For each implicated gene, we compared the CSig3 and iHRD frequency in tumors with biallelic alterations against the HRR-Reference group after removing tumors with alterations in the gene of interest from the Reference.

*ATM* alterations were identified in 85 (20%) mPCs with the majority of events involving a single copy loss (*n* = 65; 77%). Of the 15 tumors with biallelic events, *ATM* was altered in 3 by biallelic copy loss, 10 by combined mutation and copy loss/LOH, and 2 by biallelic mutation, and these included 4 with a pathogenic germline mutation. One of 15 tumors with *ATM*-BAL was CSig3(+) (7%) and 3 were iHRD(+) (20%). The CSig3(+) *ATM*-BAL tumor also had a *CHEK2* truncating kinase domain mutation. Overall, no tumor with *ATM*-BAL alone exhibited a CSig3(+) DNA mutation profile (Figure 1C and Supplemental Figure 1). Tumors with biallelic *CHEK2* loss (*n* = 6), mutations in genes involved in NHEJ repair (*n* = 11), biallelic loss in base excision pathway genes, or loss in other DNA repair-associated genes including *TP53BP1*, *MUS81*, and *REV7* were uniformly CSig3(–) and iHRD(–).

*CDK12* mutations are associated with a genomic pattern of tandem duplications in multiple cancer types, including mPC (33). Ovarian carcinomas with *CDK12* loss have reduced expression of multiple HRGs, and *CDK12* loss is reported to exhibit synthetic lethality with PARPi (34), though early results of PARPi in *CDK12*-mutant mPC have identified few responses (15, 16). Of the 418 tumors evaluated, 21 were biallelic for *CDK12* inactivation. After excluding 1 tumor with biallelic losses of *BARD1*, *FANCF*, and *FANCL*, and a second tumor with biallelic *FANCM* loss, 1 of 19 *CDK12*^–/–^ tumors were CSig3(+) and 3 were iHRD(+); frequencies were not significantly greater compared to the cohort of HRR-Reference tumors (*P* = 0.69 and *P* = 1.0, Fisher’s exact test [FES], respectively).

The loss of multiple *RNASEH2* genes was recently determined to sensitize cells to PARP inhibition with mCRPCs notable for the codeletion of retinoblastoma transcriptional corepressor 1 and *RNASEH2B* in a substantial fraction of tumors (32). We assessed the *RNASEH2B* locus in the SU2C cohort and identified 26 tumors (6%) with deep *RNASEH2B* genomic loss. Of these 26 tumors, 11 had a concurrent HRG alteration or other confounding genomic event (Supplemental Table 1). Of the remaining 15 *RNASEH2B*^–/–^ tumors, 3 were classified as CSig3(+) (20%) and 6 as iHRD(+) (40%); frequencies were not significantly greater than the CSig3 and iHRD rates in the HRR-Reference group (*P* = 0.26 and *P* = 0.08, FES, respectively).

Genomic loss of *CHD1* on Chr5q, and mutations in *SPOP*, commonly co-occur and represent a distinct subtype of mCRPC (35, 36). *CHD1* loss and *SPOP* mutations have each been shown to regulate aspects of HRR in preclinical models (8–10). Of 22 tumors with biallelic *CHD1* loss (*CHD1^–/–^*), 11 also harbored an *SPOP* mutation (*SPOP^Mut^*). Excluding tumors with concurrent HRR-modulating or -confounding events (e.g., hypermutation), the frequency of CSig3(+) tumors was significantly higher in *CHD1^–/–^ SPOP^WT^* tumors (43%; *P* = 0.02, FES; Figure 1C) but not in *CHD1^–/–^ SPOP^Mut^* tumors (0%; *P* = 1.0, FES) compared with the HRR-Reference. Though the frequency of iHRD(+) was not greater in either CHD1^–/–^ group compared to the HRR-Reference, the LOH scores of CHD1^–/–^ tumors were significantly greater compared with CHD1^+/+^ tumors (0.16 ± 0.07 versus 0.10 ± 0.08; *q* = 0.03; Supplemental Table 2). Of 26 tumors with *SPOP* mutation, *CHD1*-BAL also occurred in 11, monoallelic *CHD1* copy loss occurred in 8, and 7 had no *CHD1* alterations. Excluding tumors with concurrent HRR-modulating events, the CSig3(+) and iHRD(+), frequencies in *CHD1^+/+^ SPOP^Mut^* tumors were not significantly different compared to the HRR-Reference cohort (*P* = 0.29 and *P* = 1.0, FES, respectively).

### Multiple monoallelic HRR gene losses and low HRR gene expression are associated with HRRd mutational signatures.

**Figure 3:**
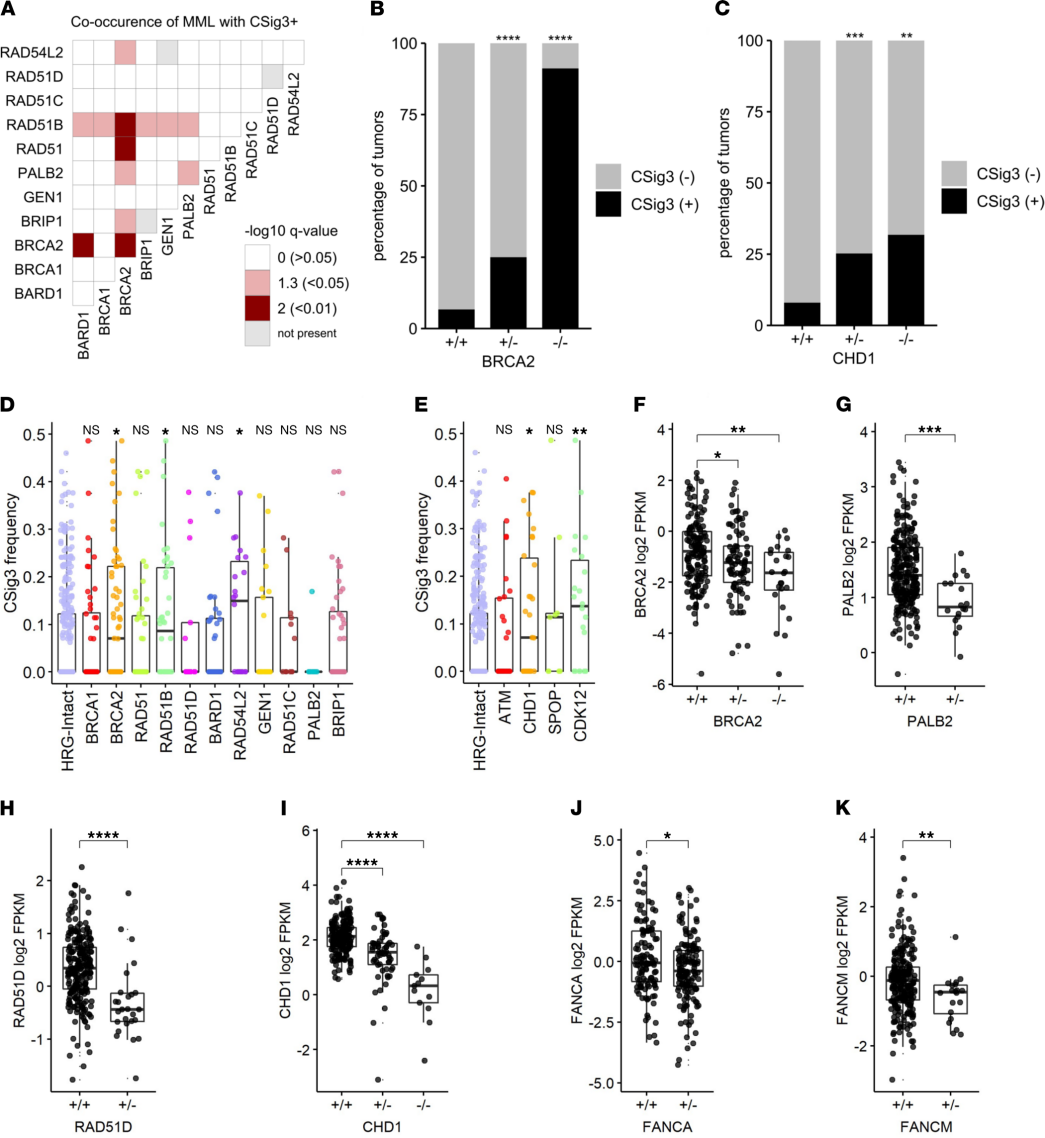
Genomic alterations and gene expression associate with HRR deficiency mutational signatures. (**A**) Assessment of co-occurrence of monoallelic aberrations in HRGs and tumors classified as CSig3(+). Fisher’s exact tests of MML co-occurrence (or BAL for same gene pairs on the diagonal) versus CSig3 status for 418 tumors. Odds ratios were positive for all significant associations; Benjamini-Hochberg–corrected *P* value significance level color-coded as red (*P* < 0.01), pink (*P* < 0.05), or white (*P* > 0.05). Gray boxes indicate gene pairs with no co-occurrence of MML or no BAL for same gene pairs. (**B** and **C**) Association of genomic loss of (**B**) *BRCA2* or (**C**) *CHD1* with CSig3 status. Comparison of heterozygous (+/–) or homozygous (–/–) loss to WT (+/+) by Fisher’s exact test in 418 tumors with Benjamini-Hochberg multiple-testing correction shown on plot (FDR * ≤ 0.05; ** < 0.01; *** < 0.001; **** < 0.0001). (**D** and **E**) CSig3 score is elevated in tumors with low transcript levels of specific genes involved in HRR. CSig3 frequencies of samples with gene expression less than 2-fold below median to the HRG-Intact group in 379 tumors by 1-sided Wilcoxon rank test with Benjamini-Hochberg multiple-testing correction shown on plot (FDR * ≤ 0.05; ** < 0.01; *** < 0.001; **** < 0.0001; NS > 0.05). (**F**–**K**) Association between genomic status of *BRCA2*, *PALB2*, *RAD51B*, and *RAD51C* and transcript expression levels. Comparison of heterozygous (+/–) or homozygous (–/–) loss to WT (+/+) in 259 tumors by Wilcoxon rank test with Benjamini-Hochberg multiple-testing correction shown on plot (FDR * ≤ 0.05; ** < 0.01; *** < 0.001; **** < 0.0001). FPKM, fragments per kilobase million.

Of the CSig3(+) tumors that did not have biallelic loss of a core HRG or FA gene, 17 (41%) had more than 1 HRG-MAL, designated as HRG-MML, and all were iHRD(+) (Figure 1, A and B). HRG-MML tumors were enriched for CSig3(+) status compared with tumors without HRG alterations (*P* < 0.05) (Figure 1C), and several combinations of genes with monoallelic loss were associated with CSig3(+) and iHRD(+) tumors (Figure 3A and Supplemental Figure 2). A higher percentage of tumors with monoallelic loss of *BRCA2* and *CHD1* were classified as CSig3(+) and iHRD(+) compared with intact tumors (*P* < 0.001) (Figure 3, B and C, and Supplemental Figure 2), suggesting that HRG haploinsufficiency may contribute to HRRd in certain circumstances (37, 38).

Studies of breast carcinoma have determined that reduced expression of HRR genes, such as *BRCA1* and *RAD51C*, primarily via promoter methylation, associates with mutational signatures of HRRd (20). We quantitated transcript levels of the core HRGs, members of the FA pathway, and other genes involved in HRR from 259 tumors where matched RNA-Seq was successful. Tumors with low expression levels of *BRCA2*, *RAD51B*, *RAD54L2*, *CHD1*, and *CDK12* were each associated with high CSig3 activity (*P* < 0.05; Figure 3, D and E).

As the CSig3 fraction is a component of the iHRD classifier, the iHRD results were in general agreement with CSig3 associations. Of the iHRD(+) tumors, 63% (*n* = 72) lacked biallelic loss of a core HRG. Several combinations of genes with monoallelic loss were associated with iHRD(+) tumors, and a higher percentage of tumors with monoallelic loss of *BRCA2* and *CHD1* were classified as iHRD(+) compared with those without alterations in these genes (*P* < 0.05; Supplemental Figure 2).

Several oncogenic mechanisms may influence gene expression, including both epigenomic and genomic events. As noted above, a substantial number of tumors were found to have loss of a single copy of a core HRG or FA gene, a subset of which also were classified as CSig3(+) and iHRD(+) (Figure 1, A and B, and Figure 2G). We next evaluated the relationship between genomic copy loss and gene expression for the core HRGs and found significantly lower transcript levels in tumors with monoallelic loss of *BRCA2* (*P* < 0.05), *PALB2* (*P* < 0.001), and *RAD51D* (*P* < 0.0001) compared with tumors without genomic alterations (Figure 3, F–H). Genomic copy loss of *CHD1* also associated with reduced CHD1 expression (Figure 3I), as did several FA genes (Figure 3, J and K; and Supplemental Figure 2, D–H). Due to lack of sample availability, we were not able to determine if methylation associated with transcript levels of these or other HRR-associated genes in these tumors.

### iHRD classification associates with functional assessments of HRRd and responses to platinum chemotherapy.

The classification of HRRd has clinical utility in selecting patients for therapies that target HRR incompetence either by exploiting synthetic lethality using PARPi or by inducing irreparable DNA damage such as that produced by platinum-based chemotherapy. Standard treatments for mPC currently center on androgen receptor (AR) pathway antagonists and taxane-based chemotherapy, neither of which explicitly target HRRd. We evaluated CSig3 activity and iHRD status in men with mPC and found no association with overall survival or survival after initiating AR signaling inhibitors (Supplemental Figure 3). Recent studies and the consequent approval of PARPi for a subset of mPCs with a spectrum of HRR-associated gene mutations indicate that the selective use of these agents provides clinical benefit in this subgroup (15, 39). However, studies of breast and ovarian carcinoma indicate that ascertainment of a core HRG mutation lacks sensitivity and specificity with respect to treatment outcomes (19, 28), and the clinical trial results of PARPi in mPC clearly demonstrate suboptimal prediction of clinical responses based on the ascertainment of HRR gene mutations (15, 39).

**Figure 4:**
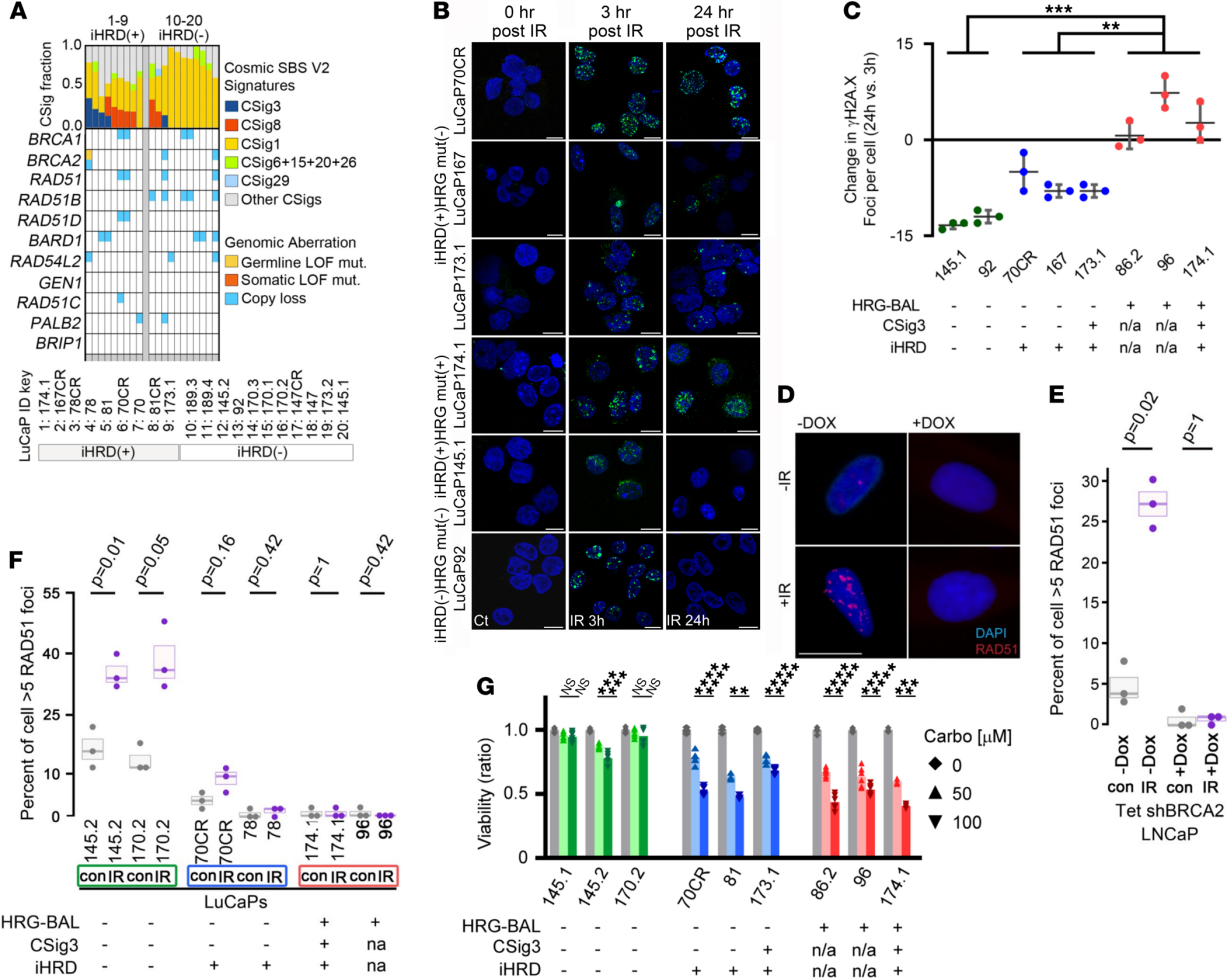
Evaluation of HRR gene mutation, COSMIC signature 3, and integrated assessment of HRR deficiency classification with responses to DNA-damaging therapeutics. (**A**) Distribution of COSMIC single base substitution mutational signatures (CSigs) across PC patient-derived xenograft (PDX) lines. The classes of COSMIC mutation signatures are color-coded, and tumors are ordered in decreasing frequency of CSig3. (**B** and **C**) Confocal immunostaining and quantitation of γH2AX foci in short-term cultures of the indicated PDX line 3 hours and 24 hours after exposure to 6 Gy IR or sham treatment (Ct). Foci counts for each time point are the mean ± SD of 3 replicate experiments. Intergroup comparison in change of foci count per cell was performed by a Welch’s *t* test. (*P* value ** < 0.01; *** < 0.001). Scale bar: 10 µm. (**D** and **E**) Immunofluorescence microscopy quantitation of RAD51 foci in LNCaP_Tet_shBRCA2 with or without DOX and with or without IR; (**D**) representative images of cells with and without designated treatments; (**E**) percentage of 50 cells counted with more than 5 RAD51 foci/cell. Each measurement represents the mean ± SD of 3 independent measurements. Significance was determined by Fisher’s exact test. Scale bar: 10 μm. (**F**) Quantitation of RAD51 immunofluorescence foci count in short-term cultures of LuCaP PDX lines exposed to IR or sham treatment. The percentage of 50 cells counted with more than 5 RAD51 foci/cell is plotted with each measurement representing the mean ± SD of 3 independent measurements. Significance was determined by Fisher’s exact test. (**G**) Cell viability assessments of LuCaP PDX lines grown in vitro and measured 72 hours after treatment with vehicle or 50 μM or 100 μM carboplatin. Each measurement represents the mean ± SD of 3 (LuCaP81 and LuCaP174.1) or 6 (remainder) replicate experiments. Comparison of cell viability was performed using paired *t* tests with Bonferroni’s corrections (*P* value * ≤ 0.05; ** < 0.01; *** < 0.001; **** < 0.0001).

To assess the potential clinical use of CSig3 and iHRD classification in treatment selection, we carried out functional studies to compare responses based on a core HRG mutation (HRGmut), CSig3 activity, and iHRD status. Using WES, we annotated a panel of 20 PC patient-derived xenograft (PDX) lines according to alterations in the core HRGs: HRG-BAL, *n* = 1; HRG-MML, *n* = 7; and HRG-MAL or WT, *n* = 12. Two lines were CSig(+), and 9 were classified as iHRD(+) (Figure 4A). Of the 9 iHRD(+) tumors, 1 was explained by *BRCA2*-BAL, 2 were HRG-MML, 3 were HRG-MAL, and 3 had no genomic alteration in any core HRG. Two iHRD(+) PDX lines were CSig3(+), and of the iHRD(–) tumors, none was CSig3(+).

We established several of the PDX lines as short-term in vitro cultures and used them to assess the function of DNA repair competency by exposing them to γ irradiation (IR) and measuring DNA double-strand breaks by quantitating γH2AX foci by immunofluorescence staining. Three hours after radiation all lines had substantially greater numbers of γH2AX foci compared with baseline, and there were no significant differences based on genotype, CSig3, or iHRD status (Figure 4B). However, 24 hours after IR, 2 lines, LuCaP92 and LuCaP145.1, classified as HRGmut(–) CSig3(–) iHRD(–), had completely resolved these foci to levels equivalent to baseline (Figure 4, B and C). Two HRGmut(+) lines, LuCaP96 and LuCaP86.2, where CSig3 and iHRD status could not be determined because of lack of germline control to ascertain mutation signatures, and 1 line, LuCaP174.1, classified as HRGmut(+) CSig3(+) iHRD(+), had persistently elevated γH2AX foci counts at 24 hours equal to or greater than foci counts at 3 hours. Three lines lacked genomic alterations in core HRR or FA pathway genes but were classified as iHRD(+): 2 of these lines, LuCaP173.1 and LuCaP167, had reduced γH2AX foci by 24 hours, though they remained significantly higher than baseline levels (*P* < 0.01) and higher than iHRD(–) lines, while foci numbers in the HRGmut(–) CSig3(–) iHRD(+) LuCaP70CR cells were the highest of all lines at 3 hours and remained substantially elevated at 24 hours (*P* < 0.001; Figure 4C).

In the setting of intact HRR, the detection of RAD51 foci by immunofluorescence microscopy following DNA damage serves as functional readout of HRR proficiency (40, 41). We next assayed RAD51 foci formation following IR exposure in PDX cell line models classified as iHRD(+) or iHRD(–). As a control, we engineered LNCaP cells to express a doxycycline-inducible (DOX-inducible) shRNA targeting BRCA2. Following IR, the number of control shBRCA2 cells with RAD51 foci increased from a baseline of 10% to 55% (*P* = 0.02) whereas the number of DOX-treated shBRCA2 cells with RAD51 foci did not change significantly (*P* = 1; Figure 4, D and E). In short-term cultures of LuCaP PDX lines, RAD51 foci were induced significantly by IR treatment in both lines classified as CSig3(–) and iHRD(–), but not in either of the CSig3(–) iHRD(+) LuCaP70CR and LuCaP78 lines, or in the HRG-BAL LuCaP174.1 and LuCaP96 lines (Figure 4F).

Tumor cells with incompetent HRR exhibit enhanced sensitivity to DNA cross-linking agents, such as platinum chemotherapy. We quantitated the viability of the LuCaP cells in vitro following 72 hours of exposure to either 50 μM or 100 μM carboplatin. The 3 HRGmut(–) CSig3(–) iHRD(–) lines, LuCaP145.1, LuCaP145.2, and LuCaP170.2, were largely resistant to both carboplatin concentrations while the HRGmut(+) lines, LuCaP86.2, LuCaP96, and LuCaP174.1, showed substantial dose-dependent reductions in viability (Figure 4G). Notably carboplatin treatment significantly reduced the viability of each PDX line classified as iHRD(+) but lacking biallelic mutations in core HRGs: LuCaP70CR, LuCaP81, and LuCaP173.1 (Figure 4G).

**Figure 5:**
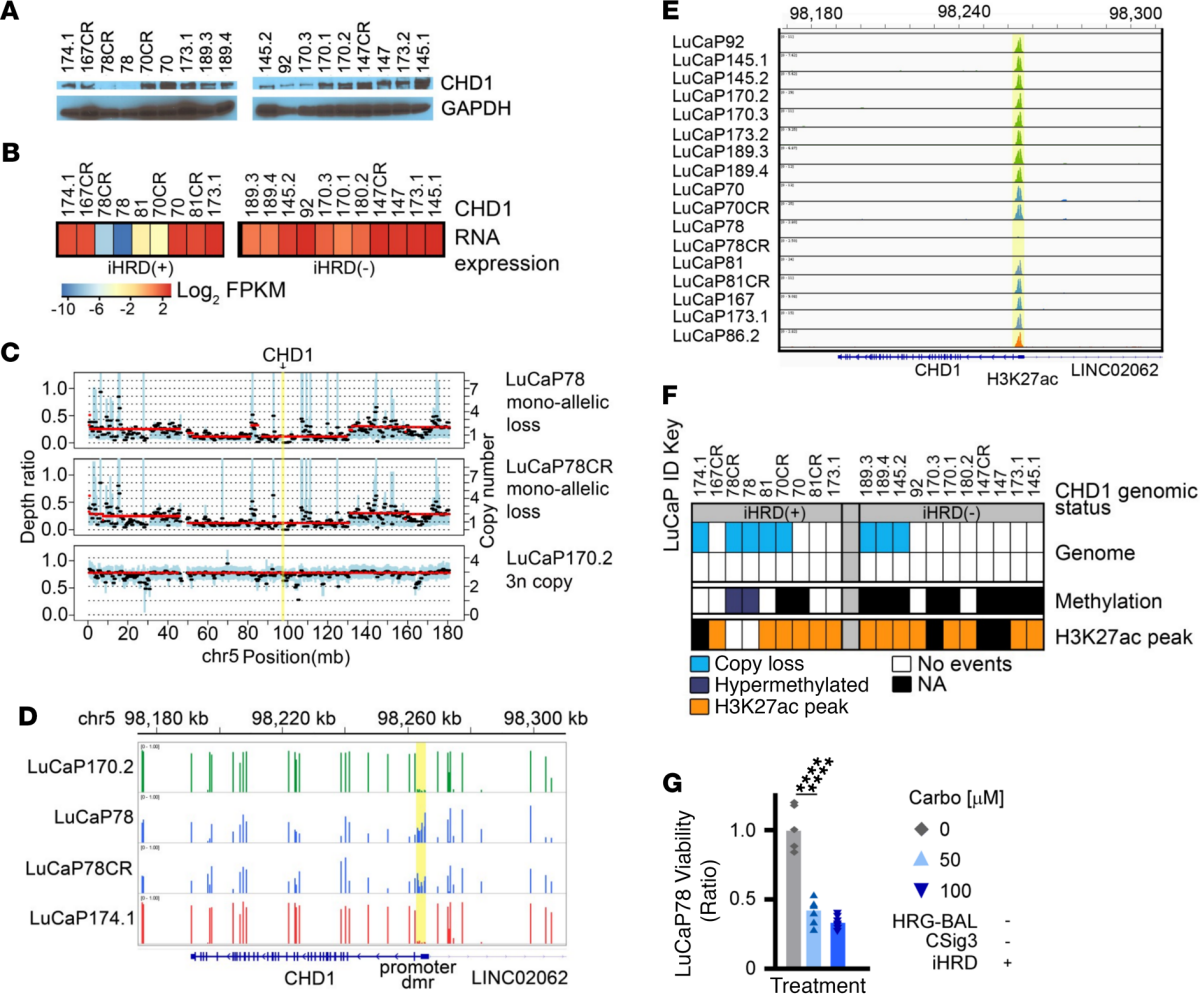
A de novo model of *CHD1* loss demonstrates functional HRR deficiency. (**A**) Western blot showing CHD1 protein expression across 20 LuCaP PC PDX lines. (**B**) Heatmap showing median centered CHD1 mRNA expression across 20 LuCaP PDX lines determined by RNA-Seq. The heatmap scale represents log_2_-scaled normalized CHD1 expression. (**C**) Copy number profile of *CHD1* in 3 representative LuCaP PDX lines determined by whole-exome sequencing. LuCaP78 and LuCaP78CR exhibit monoallelic copy number loss of *CHD1*. (**D**) *CHD1* genomic methylation status determined by EPIC methylation arrays. Shown are the IGV tracks indicating promoter hypermethylation at the upstream promoter of *CHD1* in LuCaP78 and LuCaP78CR PDX lines. A comparison of normalized beta values across probes for 12 CPG loci in the putative *CHD1* promoter region was done using a 2-sided independent-sample *t* test (*P* = 2.17 × 10^–10^). (**E**) IGV track showing the H3K27 acetylation (H3K27ac) mark at the *CHD1* 5′ promoter locus demonstrating loss of the H3K27ac peak in the upstream promoter of LuCaP78 and LuCaP78CR PDX lines. (**F**) Heatmap showing *CHD1* genomic status across 20 LuCaP PDX lines. LuCaP78 and LuCaP78 CR have *CHD1* monoallelic copy loss paired with *CHD1* promoter hypermethylation and loss of H3K27ac peak. (**G**) Cell viability assessments of LuCaP78 PDX lines grown in vitro and measured 72 hours after treatment with vehicle or 50 μM or 100 μM carboplatin. Measurement represents the mean ± SD of 8 replicate experiments. Comparison of cell viability was performed using 2-sided paired *t* tests. (*P* value: **** < 0.0001). See uncut gels in Supplemental Figure 7 and in online supplemental material.

In the analyses of mPCs, a substantial fraction of tumors without biallelic loss of a gene classically associated with HRR were classified as CSig3(+) or iHRD(+), and this group included tumors with biallelic *CHD1* loss or low CHD1 expression (Figure 1C, Figure 3E, and Supplemental Figure 2). To determine if *CHD1* loss could underlie CSig3 or iHRD classification of any of the PDX models, we measured CHD1 protein by immunoblot and identified 2 iHRD(+) lines, LuCaP78 and LuCaP78CR, that lacked CHD1 protein and CHD1 transcripts (Figure 5, A and B). Both lines, originating from the same patient, had monoallelic loss of *CHD1* determined by WES (Figure 5C). We next sought to determine if the remaining allele was silenced by methylation using Infinium methylation EPIC array hybridization and found a region of hypermethylation located in the 5′-UTR of *CHD1* (Figure 5D), which was accompanied by loss of H3K27 acetylation marks ascertained through a recent study of genome-wide H3K37 acetylation in these PDX lines (Figure 5E and ref. 42). We supported the hypermethylation status of *CHD1* in the LuCaP78 and LuCaP78CR PDX lines using a targeted methylation PCR assay and concluded that *CHD1* was lost in these tumors by a combination of monoallelic genomic loss and epigenetic silencing of the remaining allele (Figure 5F). Notably, following IR treatment, the LuCaP78 PDX cells failed to form RAD51 foci (Figure 4F), indicating functional HRRd as reflected by iHRD(+) classification, and these cells were sensitive to carboplatin treatment, with significant reductions in cell viability with both 50 μM and 100 μM concentrations (Figure 5G).

**Figure 6:**
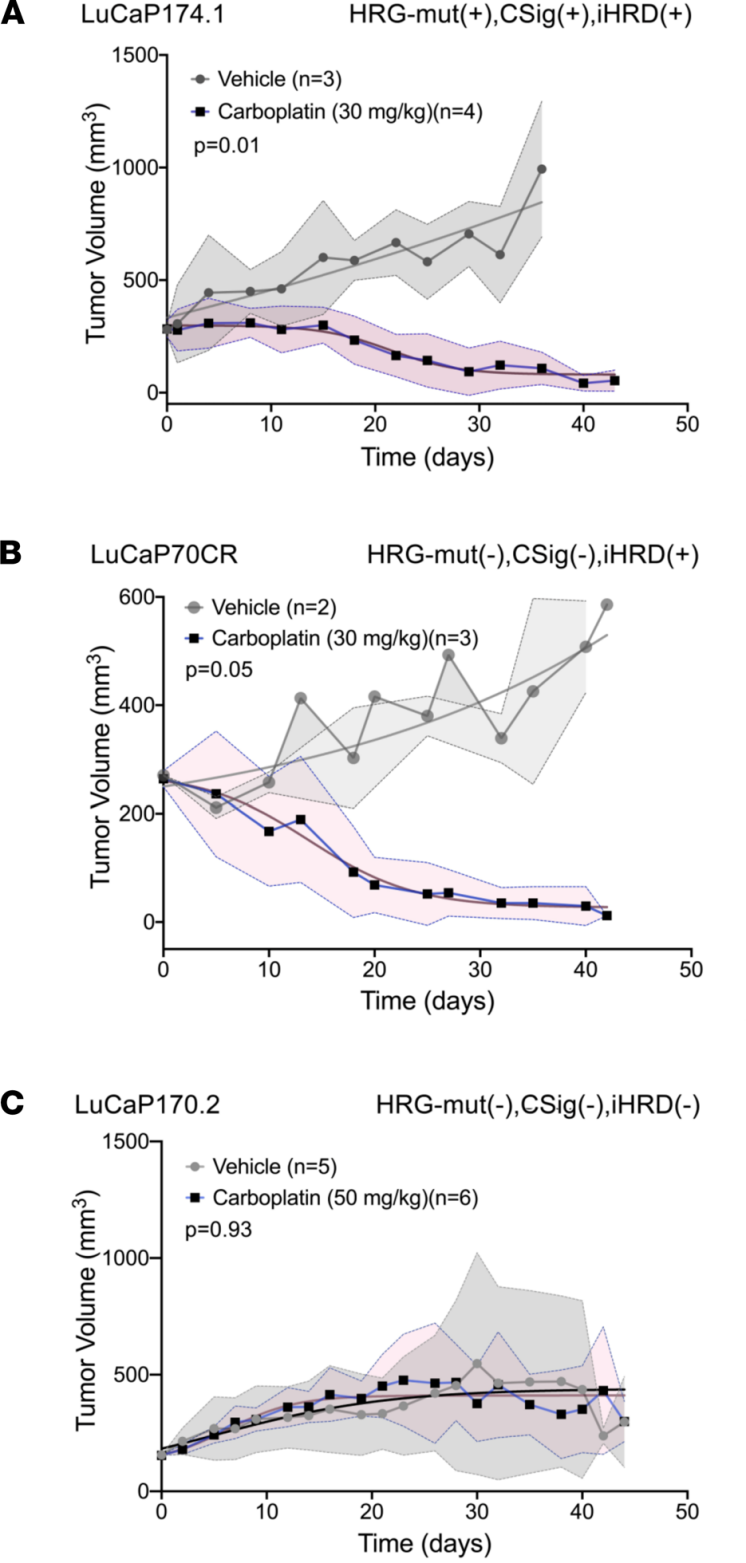
Carboplatin treatment responses associate with COSMIC signature 3 and integrated assessment of HRR deficiency classification in patient-derived xenograft models. (**A**–**C**) LuCaP PDX TVs measured over time in mice treated with carboplatin 30 mg/kg or vehicle control IP weekly. At a TV of approximately 150 mm^3^ mice were randomized to treatments. Carboplatin significantly reduced the growth of iHRD(+) lines compared with vehicle with end-of-study LuCaP167 volumes of 42 ± 6 vs. 615 ± 288 (*P* < 0.01) and LuCaP70 end-of-study volumes of 857± 206 vs. 38 ± 7 (*P* < 0.01). Carboplatin did not affect the growth of iHRD(–) LuCaP 170.2 compared to vehicle treatment (*P* = 0.11). In all plots, each time point is represented as mean tumor volume ± SD. Tumor growth curve comparisons were determined by permutation tests.

We next assessed carboplatin effects on iHRD(–) and iHRD(+) PDX lines in vivo. PDX tumors were implanted in immunodeficient mice, allowed to grow to approximately 150 mm^3^, and then randomized to vehicle control or weekly carboplatin. The HRGmut(+) CSig3(+) iHRD(+) LuCaP174.1 line demonstrated a substantial response with end-of-treatment tumor volumes (TVs) of 42 ± 34 mm^3^ with carboplatin versus 994 ± 302 mm^3^ with vehicle (*P* = 0.01; Figure 6A). Two iHRD(+) lines without mutations in core HRR genes, LuCaP70 and LuCaP167, also exhibited exceptional responses to carboplatin, with substantial differences in end-of-treatment TVs (*P* < 0.03; Figure 6B and Supplemental Figure 4). Treatment had no or modest effects on the growth of the HRGmut(–) CSig3(–) iHRD(–) LuCaP170.2 or 145.1 lines, even with higher carboplatin concentrations (*P* = 0.93; Figure 6C and Supplemental Figure 4).

### Mutation signatures and iHRD classification associate with clinical responses to therapeutics exploiting HRRd.

**Figure 7:**
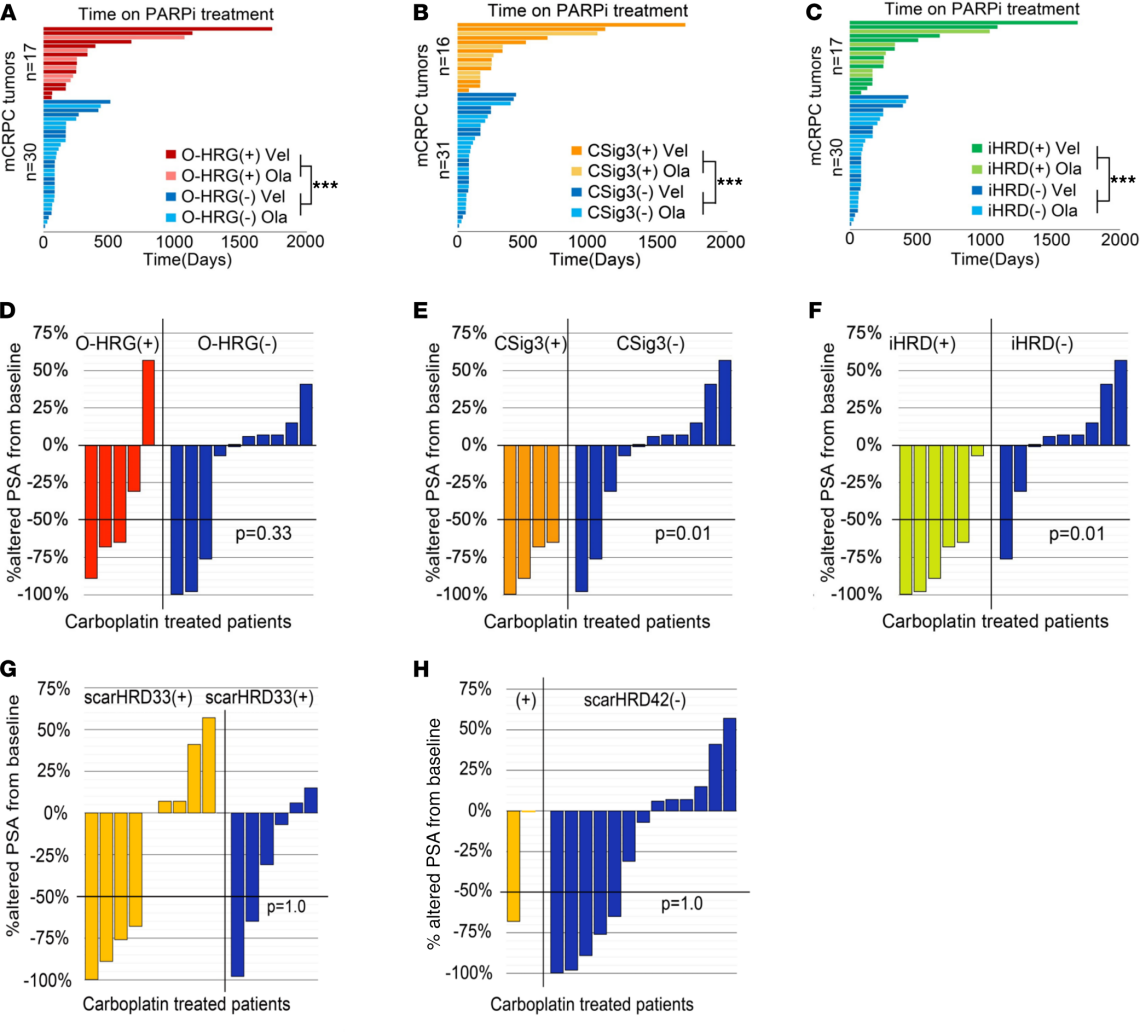
Clinical treatment outcomes associate with tumor COSMIC signature 3 and integrated assessment of HRR deficiency classification. (**A**–**C**) Clinical outcomes of PARPi treatment shown by time (days) on drug depicted in swimmer plots partitioned by: (**A**) HRG mutation status: tumors are classified as O-HRG(+) or O-HRG(–) based on the biallelic loss of 1 of 14 genes approved for olaparib treatment; (**B**) CSig3 classification; (**C**) iHRD classification. (**D**–**H**) Clinical outcomes of carboplatin chemotherapy shown by the maximum decline in serum prostate-specific antigen measurements. Tumors are classified by (**D**) O-HRG(+) or O-HRG(–) based on biallelic loss of 1 of 14 genes approved for olaparib treatment; (**E**) CSig3 classification; (**F**) iHRD classification; (**G**) scarHRD classification using a score ≥33 as positive for HRRd; (**H**) scarHRD classification using a score ≥42 as positive for HRRd. Comparison of time on PARPi treatment was performed using Mann-Whitney *U* test, and associations of biomarker status to carboplatin PSA50 response was determined using Fisher’s exact test (*P* value *** < 0.001).

To further assess the relationship between aberrations in genes involved in HRR, HRRd-associated mutational signatures, and iHRD classification with treatment responses, we retrospectively evaluated patients in the SU2C cohort treated with PARPi. We identified 47 patients who received PARPi, of which 23 were treated with olaparib and 24 were treated with veliparib. Based on the PROFOUND clinical trial (15), olaparib is approved for men with mCRPC and a pathogenic mutation in *ATM*, *BRCA1*, *BRCA2*, *BARD1*, *BRIP1*, *CDK12*, *CHEK1*, *CHEK2*, *FANCL*, *PALB2*, *RAD51B*, *RAD51C*, *RAD51D*, or *RAD54L*, collectively, olaparib-approved HRG mutations: O-HRGmut(+). We annotated each tumor as O-HRGmut(+) only if biallelic pathogenic events were identified or if a germline mutation was present. Overall, tumors from 16 PARPi-treated patients were O-HRGmut(+), of which 3 had germline mutations, 16 were CSig3(+), and 17 were classified as iHRD(+), including all CSig3(+) tumors. As objective response data were not available, we used time on PARPi treatment as a surrogate for treatment efficacy. All 3 HRRd predictors were strongly associated with longer time on PARPi treatment, though with modestly different discrimination with respect to treatment duration: 440 versus 141 days for O-HRGmut(+) versus O-HRGmut(–) (*P* < 0.001); 467 versus 137 days for CSig3(+) versus CSig3(–) (*P* < 0.001), and 447 versus 138 days for iHRD(+) versus iHRD(–) tumors (*P* < 0.001; Figure 7, A–C). We also evaluated each tumor based on other genomic metrics indicative of HRRd that include telomere allelic imbalance, loss of heterozygosity, and large-scale state transitions, collectively termed genomic scars. Measures of these parameters are combined to generate an overall score, scarHRD, with a cut point more than 42 associated with HRRd and treatment responses in breast carcinomas (27) and a cut point more than 33 associated with PARPi responders in ovarian carcinoma (43). Unlike O-HRGmut status, or CSig3 or iHRD classification, scarHRD cutpoints of more than 42 or more than 33 did not significantly discriminate time on PARPi (Supplemental Figure 5 and Supplemental Table 5).

Though formal prospective studies are lacking, patients with PC with *BRCA2* mutations have demonstrated exceptional responses to carboplatin therapy (12, 13). As the SU2C study did not consistently capture platinum treatment, we evaluated responses in the University of Washington rapid autopsy cohort (Supplemental Figure 6). Of 88 patients, 15 were treated with carboplatin. We classified these tumors according to O-HRG(+) (*n* = 5), CSig3(+) (*n* = 4), scarHRD > 33 (*n* = 8), scarHRD > 42 (*n* = 2), and iHRD(+) (*n* = 6). A decline in serum prostate-specific antigen levels of 50% or greater (PSA50) has been used as an objective measure of treatment response (44). Three of 5 O-HRG(+) and 3 of 10 O-HRG(–) patients achieved a PSA50, indicating that these biomarkers lack high sensitivity or specificity (Figure 7D). Similarly, classification by scarHRD also lacked sensitivity or specificity for carboplatin responses (Figure 7, G and H), suggesting that the features or scarHRD cut points used for classifying breast and ovarian carcinoma may not appropriately capture HRRd status in prostate tumors. The sensitivity and specificity for CSig3 status predicting PSA50 responses to carboplatin were 66% and 100%, respectively (Figure 7E), whereas the sensitivity and specificity of iHRD in predicting carboplatin responses were 83% and 89%, respectively (Figure 7F).

## Discussion

Cancers of the breast, ovary, pancreas, and prostate are each associated with relatively high frequencies of germline and/or somatic mutations in *BRCA1/2* resulting in HRRd (45). Congruent with concepts underlying precision medicine, cancers with these mutations exhibit high response rates to treatments that exploit HRRd, such as platinum-based chemotherapy and PARPi. However, clinical trials of these drugs have demonstrated enhanced clinical benefit in subsets of patients without *BRCA1/2* mutations (28, 46). Ongoing studies designed to evaluate additional genes involved in the HRR pathway have determined that alterations in *PALB2* and *RAD51B* may associate with responses whereas several genes hypothesized to confer benefit, such as *CDK12* and *ATM*, have not consistently been correlated with enhanced benefit (14–16). Further, in addition to gene inactivation by mutation, mechanisms involving gene expression silencing by methylation can serve as a second hit and confer HRRd, as shown for *BRCA1* and *RAD51C* (20). Collectively, though the causal events underlying HRRd are becoming clearer, comprehensive testing for every possible mechanism remains challenging.

An alternative biomarker involves quantifying the genomic consequences of HRRd regardless of the specific mechanism that underlies loss of HRR proficiency. Analyses of whole-genome sequences from tumors with pathogenic *BRCA1* or *BRCA2* loss identified multiple types of associated genomic aberrations that include base substitution patterns, specifically defined as SBS signature 3; deletions with microhomology, rearrangements involving deletions < 1 Mb; and widespread loss of heterozygosity (17, 18). Integrating these genomic features into a score termed HRDetect demonstrates a very high sensitivity for HRRd associated with *BRCA1/2* loss in breast cancers. Further, about one-third of breast, ovarian, and pancreatic cancers with high HRDetect scores lack a clear underlying defect in *BRCA1/2* (19).

In the present study we used WES as an alternative to whole-genome sequencing (WGS) to determine the spectrum of mutational processes operative in metastatic PC. In view of the therapeutic importance, we focused on determining the frequency of HRRd, sought to identify features beyond *BRCA1/2* contributing to HRRd, and assessed whether HRRd features associate with responses to therapies designed to exploit HRRd. WES-based derivation of SNV mutation signatures determined that 84% of tumors with germline or somatic biallelic loss of *BRCA1/2* exhibited high CSig3 scores whereas 89% of these tumors were iHRD(+), indicative of HRRd. Notably, 10 of 55 (18%) tumors with a germline mutation in a core HRG accompanied by somatic loss of the second allele, or biallelic somatic loss, did not exhibit a mutational pattern indicative of HRRd, and each of these tumors also was classified as negative using the multiparameter iHRD algorithm. We speculate that, lacking the genomic consequences indicative of HRRd, these tumors would not be sensitive to platinum or PARPi therapy and may explain a subset of cases that exhibit primary resistance in clinical trials using HRG mutation status as selection criteria.

Alterations in *ATM*, *CDK12*, and *CHEK2* have been considered as biomarkers for PARPi response, and each is now approved for allocating olaparib therapy. However, only 3 of 15 tumors with biallelic *ATM* loss, 4 of the 19 tumors with *CDK12*-BAL, and no tumors with *CHEK2* loss were CSig3(+) or iHRD(+). A recent study reported low LOH scores in PCs with *ATM* or *CHEK2* alterations (29). These data indicate that inactivation of these genes does not generally confer genomic features of HRRd and consequently may not be strong predictors of PARPi and potentially platinum sensitivity (47). Notably, recent findings from clinical trials of rucaparib and olaparib found very limited responses in patients with *ATM*, *CDK12*, or *CHEK2* mutations (15, 16). In our retrospective cohort, the 11 patients with biallelic loss of the core HRGs *BRCA2*, *RAD51B*, and *RAD51C* were maintained on PARPi for a mean of 593 days, compared with 191 days for the 6 patients with biallelic aberrations in *ATM*, *CDK12*, or *CHEK2*. Collectively, these data indicate that alterations in these genes do not consistently result in functional HRRd, and they should not be used independently as biomarkers for treatment allocation.

Overall, of the 115 tumors called iHRD(+) by the integrated analysis of HRRd-associated genomic features, 69 (60%) lacked clear biallelic loss of a gene well established to mediate HRR, prompting further analyses of these tumors for other mechanisms contributing to HRRd. In vitro studies of *SPOP* and *CHD1* have shown that each is associated with chromosomal instability and responses to DNA-damaging agents (8–10, 48). While mechanistic details are lacking, *SPOP* and *CHD1* are reported to regulate *TP53BP1* and γH2AX recruitment to sites of double-stranded DNA breaks, with loss of function resulting in preferential NHEJ repair versus HRR, though structural genomic features congruent with functional HRRd have not been reported. Further assessments of *CHD1* for clinical associations with platinum and PARPi responses are warranted.

There remain a substantial number of mPCs with mutation spectra indicative of HRRd for which no detectable known causal event was identified. A recognized limitation of our study concerns the relatively sparse mutation data derived from WES, which also lack notable genomic features such as microhomology-flanked deletions that support HRRd classification (19, 30). Further, approaches that ascribe mutation signatures to a reference set may overfit assignments to produce false positives (49). However, prior studies using WGS of breast cancers also classified tumors as HRRd by HRDetect where no underlying HRG mutations were identified (19, 21). We hypothesize that a subset of tumors exhibit HRRd by virtue of the loss of a single allele of more than 1 gene involved in HRR, or in certain circumstances, loss of a single allele accompanied by an epigenetic event, a metabolic alteration, or environmental exposure. We determined that tumors with monoallelic loss of more than 1 core HRG were more likely to be classified as iHRD(+) compared with tumors with monoallelic loss of a single HRG or without any mutation or copy loss in these genes. Though monoallelic HRG loss has not generally been considered to impair HRR, several reports indicate that in certain contexts, for example under replication stress, HRR insufficiency occurs with single-copy *BRCA1* loss (37, 50). A study of *BRCA1/2* alterations across cancer types reported significantly higher measures of a composite HRRd score in tumors with heterozygous loss of *BRCA1/2* compared with tumors without alterations (51). While a subset of these cancers classified as heterozygous *BRCA1/2* loss may have inactivated the second allele by methylation, it is notable that environmental and endogenous genotoxins such as aldehydes have been shown to induce *BRCA2* haploinsufficiency and consequent genome instability (38, 52, 53). These findings have implications with respect to lower levels of HRR proteins conferred by single-allele loss, variation in genes regulating endogenous metabolism, and gene-environment interactions. Further analyses of tumors with functional features of HRRd without a conventional explanation, such as biallelic HRG inactivation, may uncover additional polygenic or epigenetic mechanisms influenced by internal or exogenous exposures that contribute to HRRd.

## Methods

### Study cohort.

We analyzed 418 unique tumors from the cohort of 429 reported in Abida et al. (7). We excluded any sample where the estimated tumor DNA content was less than 15%. The independent validation case set comprised 139 PC metastases acquired through the University of Washington rapid autopsy program (54). We also analyzed WES data from 20 PDX lines established from 12 individuals (55). All exomes were compared against WES data from respective paired benign tissue or blood samples where available.

### DNA sequence analysis.

WES reads were aligned to human genome reference Hg19 using BWA aligner (56). “GATK Best Practices” were followed for preprocessing BAM files (57). Unified Genotyper v3.8 was used to call germline mutations and short indels, and sSNVs were called using MuTect (version 1) (58). We included loci for variant calls that were covered by at least 14 high-quality aligned (Q40) normal reads with variant allele loci with a minimum of 6 alternate alleles. Annovar was used for annotation of all called variants. Mutalyzer 2.0 was used to validate the annotation reference. Germline pathogenic mutation annotations were performed following previously described methods (5). Somatic pathogenic mutations in DNA repair pathway genes and loss-of-function classifications were performed following the approach for classifying germline pathogenic variants and were cross-checked with annotations in cBioPortal, ExAC, Kaviar, ClinGen, Clinvar, ClinVitae, CIViC, OncoKB, and UniProt databases (Supplemental Table 6).

### Mutational signature analysis.

Mutational signatures were evaluated using the DeconstructSigs v1.7.0 installed in R release version 3.3.3 for Ubuntu Linux. The signature analyses were performed on sSNVs that passed quality filters (59). The mutation signature allocations were derived using sSNVs based on predefined CSigs. Final groups were then clustered based on mutational processes (e.g., mitotic clock/aging, APOBEC, homologous recombination defect). We considered a signature call reliable when the number of detected sSNVs for a tumor-normal pair was more than 50. For CSig3 we called a tumor CSig(+) if the CSig3 weight exceeded 0.20 of the total sSNVs and at least 50 mutations were assigned to this signature (60).

### Copy number analysis.

Sequenza was used to perform absolute copy number calling and estimation of tumor cellularity and ploidy (61).Whole-exome sequences were aligned using the BWA aligner (62). A Q20 base quality cutoff was used to avoid potential DNA alignment-linked biases. Initial segmentation calls were made using the Bioconductor “copynumber” R package, followed by Sequenza’s probabilistic model-based copy-number estimation (58, 61). Gene-restricted copy number was derived using Gencode v19 annotation references. Simultaneously, we evaluated copy number call concordance with GISTIC-derived cBioPortal calls and performed manual curation for discordant calls in HRR pathway genes.

### LOH score and ploidy determination.

Sequenza was used to perform allelic copy number calling and estimation of tumor ploidy and tumor cellularity. BWA-aligned tumor-normal paired exomes were filtered for Q40 alignment quality. To avoid bias related to genome-wide guanine-cytosine content depth, a normalization step was performed using Hg19 guanine-cytosine content data from the University of California Santa Cruz genome browser. Initial segmentation calls were made using the Bioconductor R package “copynumber” (63). Sequenza’s probabilistic model-based copy number estimation was implemented. Estimated tumor ploidy and LOH score were extracted from the analysis output score summary file, named as $tumorID.score.txt. Segment counts were extracted from the Sequenza output file $tumorID.copynumber_calls.txt.

The LOH score is defined as the ratio of the genome affected by LOH events relative to the total number of sequenced nucleotides for which the tumor copy number state can be successfully inferred. We counted toward this score events that were greater than 15 Mb in length and defined by a non-0 copy number count with an inferred minor allele count of 0 (27). Chromosomes that were affected by such LOH events over ≥75% of their entire length were excluded from both numerator and denominator, as they typically arise through non-HRD-associated mechanisms.

### iHRD status determination.

The iHRD framework (integrating HRRd-linked genomic parameters to identify tumors with HRRd) is a nonlinear classification system formulated on the foundation of a Support Vector Machines algorithm implementing a Gaussian Radial Basis Function kernel. In other words, iHRD is a binary classification model, trained to classify mCRPC tumors as HRR deficient or proficient, using WES-derived genomic features. In particular, 6 genomic features, CSig3 scores, CSig8 scores, LOH score, total number of somatic mutations, tumor ploidy, and the total number of genomic segments altered, were incorporated as features in the model to develop an approach for classifying HRRd. Method development and details are provided in Supplemental Methods.

### scarHRD determination.

scarHRD is an approach to call HRRd based on genomic parameters of telomeric allelic imbalance, LOH, and the number of large-scale transitions determined by next-generation sequencing — either WES or WGS (64). scarHRD is distributed as an R package (https://github.com/sztup/scarHRD). We used Sequenza-derived paired copy number analysis to generate binned.segz files for each tumor and normal sample pair. We then performed scarHRD analysis on the respective *.binned.segz.gz files. We used 2 scarHRD cut points of 33 and above and 42 and above to classify HRRd tumors.

### RNA-Seq analysis.

RNA-Seq data from the previously published SU2C/Prostate Cancer Foundation (PCF) cohort were aligned as described in Abida et al. (7). All subsequent analyses were performed in R. Gene-level abundance was quantitated using GenomicAlignments (65) and transformed to log_2_ FPKM.

### Protein analysis.

For CHD1 immunoblotting, frozen pieces of LuCaP xenografts were ground to a fine powder with a mortar and pestle under liquid nitrogen. The tissue powder was solubilized with RIPA buffer (Thermo Fisher Scientific catalog 89900) containing protease inhibitors (Roche catalog A32953). DNA in the lysates was sheared by sonication and the insoluble material removed by centrifugation for 10 minutes at 10,000*g*. Protein lysates (20 μg) were run on NuPAGE 4% to 12% Bis-Tris gels (Thermo Fisher Scientific catalog NP0342) with NuPAGE MOPS SDS buffer (Thermo Fisher Scientific catalog NP0001-01) and transferred to nitrocellulose membranes (Thermo Fisher Scientific catalog LC2001). Membranes were blocked with PBS containing 0.1% Tween 20 and 5% milk for 1 hour. The membranes were then probed overnight at 4°C for CHD1 (Cell Signaling Technology catalog 4351), 1:1000 dilution, and GAPDH (GeneTex catalog GTX627408), 1:5000 dilution, in PBS/0.1% Tween 20 and 2% milk, followed by a 1-hour incubation in HRP-labeled secondary antibody (Thermo Fisher Scientific catalog PI-31430), 1:10,000 dilution. Signal was detected using SuperSignal West Pico Plus Chemiluminescent Substrate (Thermo Fisher Scientific catalog 34580).

### DNA damage assessments and methylation analyses.

Assays for γH2AX and RAD51 foci were performed on freshly harvested PDX tissues and cell lines. Details of methods are provided in Supplemental Methods. Genome-scale methylation analyses were carried out using Infinium MethylationEPIC BeadChip arrays (Illumina), and *CHD1* locus-specific DNA methylation analyses were performed using COMPARE-MS (66). Details of methods are provided in Supplemental Methods.

### Drug response assays.

For drug dose response assays, 8000 cells were plated in each well of 96-well, clear, flat-bottom plates. Drugs were serially diluted in base media and applied to a total volume of 100 μL per well. After 4 days of culture, viability was assessed by the addition of 30 μL/well of CellTiter-Glo (Promega, catalog G7572) and measuring luminescence on a Synergy H1 microplate reader (BioTek, Agilent Technologies). Carboplatin (MilliporeSigma, C2538) stock solution for all in vitro experiments was prepared with Invitrogen UltraPure distilled water.

### In vivo PDX studies.

Male NSG mice (*n* = 24 per PDX) were implanted subcutaneously with approximately 3 to 5 mm^3^ tumor tissue. We used 5 LuCaP lines (LuCaP70CR, LuCaP167, LuCaP145.1, LuCaP174.1, and LuCaP170.2, derived from patients in-house) based on their HRG mutation status, CSig3 status, and iHRD classification status. When tumors exceeded approximately 150 mm^3^, animals were randomized to 2 treatment arms: vehicle control or carboplatin at a dose of 30 mg/kg or 50 mg/kg. Carboplatin solution was made using sterile injectable saline. Both treatment and vehicle arms were injected once per week IP. Treatments were administered for 6 weeks. TV and body weight were measured twice a week. Animals were sacrificed at 6 weeks, when tumors exceeded 1200 mm^3^, or when animal health was compromised.

### Data availability.

The WES data sets generated during and/or analyzed during the current study are available in the cBioPortal (query search term prad_su2c_2019, https://www.cbioportal.org/study/summary?id=prad_su2c_2019) and GitHub under the accession https://github.com/cBioPortal/datahub/tree/master/public/prad_su2c_2019 and commit ID c2e14e6. The ChIP-Seq data were downloaded from the Gene Expression Omnibus repository under accession number GSE161948.

### Statistics.

We used R to perform statistical analysis. Statistical analyses pertaining to each figure or table are included within appropriate legends. For direct comparison of continuous variables for 2 groups, 2-tailed Student’s *t* test for independent samples or Mann-Whitney *U* tests were utilized, while χ^2^ was employed for categorical variables as appropriate. For 2 × 2 categorical comparisons, we performed FES (*n* < 5) using the fisher.multcomp function in R with Benjamini-Hochberg multiple-testing correction. CSig3 frequencies were compared with genomic and low expression groups using 1-sided Wilcoxon rank tests with Benjamini-Hochberg multiple-testing correction. Gene expression (log_2_ FPKM) versus genomic status was compared using Wilcoxon rank tests with Benjamini-Hochberg multiple-testing correction. Co-occurrence of multiple monoallelic losses with CSig3 or iHRD was performed by pairwise FESs of all gene combinations followed by Benjamini-Hochberg multiple-testing correction. For survival analysis, a log rank test statistic was used. For in vivo tumor growth curve comparisons, we used “statmod” R package to implement permutation with the permutation test analysis module “compareGrowthCurves.” *P* < 0.05 was considered statistically significant. Graphical plots were prepared with R, and plot integration was done using Adobe Photoshop CC2019.

### Study approval.

All patients provided written informed consent and were enrolled based on protocols approved by Institutional Review Boards at the Fred Hutchinson Cancer Research Center and the University of Washington. All animal procedures were approved by the Fred Hutch Institutional Animal Care and Use Committee and performed in accordance with NIH guidelines. NOD-scid IL2gamma^null^ mice were acquired and bred in Fred Hutchinson Cancer Research Center’s Comparative Medicine facility. We used exome sequencing data from rapid autopsy tissue samples that were previously published (54).

## Author contributions

NDS, EVA, RBM, MTS, and PSN designed the study; NDS, SD, SJS, IC, BH, and EAK performed bioinformatics applications and statistical analyses; NDS, IC, GH, and CCP performed data curation and mutation analyses. CM, EC, RBM, and MTS provided biospecimens; PC, SBF, MCH, JML, RP, RFD, and NDS performed in vitro gene expression studies and drug response assays; LSA, TAN, and EC performed in vivo drug response assays; NDS and PSN drafted the initial manuscript; all authors provided discussion and feedback; PSN supervised; NDS and IC prepared figures; and all authors were responsible for the final draft and editing of the manuscript.

## Supplementary Material

Supplemental data

Supplemental tables 1-6

## Version 1 12/08/2021

Electronic publication
