# Supplementary material for: Genomic attributes of homology-directed DNA repair deficiency in metastatic prostate cancer
Source: JCI Insight. 2021 Dec 8;6(23):e152789. doi: 10.1172/jci.insight.152789 (PMC8675196; doi:10.1172/jci.insight.152789)
Supplement: Supplemental data [file jciinsight-6-152789-s068.pdf]

Supplementary Materials

Genomic attributes of homology directed DNA repair deficiency in metastatic prostate cancer

Navonil De Sarkar[1,2], Sayan Dasgupta[3], Payel Chatterjee[1,2], Ilsa Coleman[1,2], Gavin Ha[1,4], Lisa Ang[1,2], Emily Kohlbrenner[1,2], Sander Frank[1,2], Talina Nunez[1,2], Stephen J. Salipante[5], Eva Corey[6], Colm Morrissey[6], Eliezer Van Allen[7], Michael T. Schweizer[8], Michael C. Haffner[1,2], Radhika Patel[1], Brian Hanratty[1], Jared M. Lucas[1], Ruth F. Dumpit[1], Colin C. Pritchard[5], Robert B. Montgomery[8], and Peter S. Nelson[1,2,4,5,6,8*]

Author affiliations:

[1]Divisions of Human Biology, [2]Clinical Research and [3]Vaccine and Infectious Disease and [4]Public Health Sciences, Fred Hutchinson Cancer Research Center, Seattle, WA, USA

[5]Department of Laboratory Medicine and Pathology, University of Washington, Seattle, WA, USA

[6]Department of Urology, University of Washington, Seattle, WA, USA

[7]Dana Farber Cancer Institute, Boston, MA, USA

[8]Department of Medicine, Division of Oncology, University of Washington, Seattle, WA, USA

Supplementary Methods

Supplementary Figures S1-S7.

Supplementary Methods.

iHRD Development and Implementation. The iHRD (integrated analysis of homology directed repair deficiency) framework is a nonlinear classification system formulated on the foundation of a Support Vector Machines algorithm implementing a Gaussian RBF (Radial Basis Function) kernel. In other words, iHRD is a binary classification model, trained to classify mCRPC tumors as HR DNA repair deficient or proficient, using WES derived genomic features. We utilized six genomic features: CSig3 scores, CSig8 scores, LOH score, total number of somatic mutations, tumor ploidy, and the total number of genomic segments altered, in the model to develop an approach for classifying HRR deficiency. For training, 238 exomes were selected for the SVM classification. These included 48 tumors with biallelic loss of 11 core genes involved in HR DNA repair and 190 without HRR gene mutations. The criterion for non-HRRd comparator samples were: CDK12 intact, no bi-allelic loss in a NHEJ pathway gene, no CSig3 or CSig8 activity, no hypermutated genome, no *ATM* or *CHEK2* biallelic loss.

The iHRD model development and analysis involved 3 steps. *Step1: Tuning iHRD Parameters:* For training the classifier, we used data derived from 238 tumor samples described above. We adopted a 10-fold cross-validation method to define optimal cost and gamma for the classification. Model training on the 238 samples was executed using these derived optimal tuning parameters. The misclassification rate of the training set was ~2.1%. *Step2: Evaluation of Model Performance:* We used the 238 training set samples and evaluated a battery of cross-validation (CV) methods to test the performance of the trained model. Overall, the model training error was ~4%. The CV performance measures were: (a) boot strap error: 0.063; (b) bootstrap 0.632 error: 048; (c) 10-fold CV error rate: 0.053; (d) 10-fold CV precision: 0.94; (e) F measure -10-fold CV: 0.84;; and (f) AUC.ROC: 0.95. *Step3: Validation using an independent sample set*: The independent validation set comprised WES data from 89 tumors from the University of Washington rapid autopsy cohort (n=52), LuCaP PDX tumors (n=8), and SU2C tumors (n=29) not used in the original model development. The model validation performance measures were: (a)

recall rate: 0.77; (b) Precision: 0.85; (c) F measure: 0.81 and (d) misclassification error rate: 0.09. The R script for the iHRD classification is available through GitHub: https://github.com/ndesar/iHRD-Predict.R.

To determine the importance of each of the six selected iHRD features, we performed cross-validation recursive feature elimination (CV-RFE) analysis to derive the rank order of each of the 6 features used in the integrated SVM-based classification (1, 2). The ranking signifies the relative contribution to the overall iHRD classification. The derived rank is CSig3> CSig8> Number_of_segments >Mutation burden> Ploidy>LOH. Although in univariate analysis, ploidy contributed minimally to HRRd classification, it ranked higher in the iHRD full model. To note, certain features like detected Number_of_segments and estimated LOH are likely a function of estimated tumor ploidy. To understand the importance of each of these features in the model we derived model performance matrices taking one feature out in the sequence of RFE rank (CV-RFE analysis). The full 6 feature model-based analysis is named step 0, and subsequently, step 1 stands for the iHRD model excluding the least ranked feature (e.g. LOH). Based on this analysis, the best model was step 2 (full 6 feature iHRD model excluding LOH and ploidy). Although LOH is not a major contributor of HRRd predicter in mPC, LOH has a substantial association with HRRd in breast and ovarian cancer and comprises a component of other classification tools such as HRDetect developed for WGS. As iHRD may be useful in HRRd classification using WES in other tumor types, we opted to include all 6 features in the final iHRD model. Further, compared to CSig3 alone, the current iHRD model has substantially better precision.

We compared the SVM-RBF approach with an alternative generalized linear model (GLM) that accommodates response variables including those with a non-normal error distribution. For the task of classification, we used a GLM with a binomial logit link function. Using the GLM model, the misclassification rate was 0.038 with 3 false-positive predictions in the set of 238 training samples (recall =0.88; precision = 0.93; F-measure=0.90), which is higher than the SVM-RBF

misclassification rate of 0.021 with 0 false-positive annotations (recall=0.90; precision=1.00; F-measure=0.95). The misclassification error for the SVM model performance on a validation sample set was 0.090 (recall=0.77; precision=0.85; F-measure=0.81) compared to the GLM misclassification error of 0.101 (recall=0.77; precision=0.81; F-measure=0.79)

To compare GLM and SVM-RBF using the six feature iHRD parameters we performed univariate analyses comparing the SVM-RBF and GLM approaches with both default threshold-based analysis and data-driven optimal thresholds based on maximization of Youden's index. For GLM, the 3 significant or informative contributory features are: CSig3, CSig8, and genome segment count. Data curation on HRR gene mutants with respect to HRG intact tumor samples indicated that both hypermutated tumors and the tumors with low mutation burdens are enriched in the HRRd intact tumor class, leading to noisy behaviors of these features in the GLM model. With respect to accuracy and precision, nonlinear SVM metrics for LOH, mutation burden, and segment count are substantially better than GLM based measures; reconfirming rationale for choosing the nonlinear SVM as the framework for iHRD.

DNA damage assessments: γH2AX and RAD51 foci. Freshly harvested PDX tissues were immediately chopped and digested in 10% serum containing digestion media (1mg/ml collagenase+1 mg/ml dispase; 37° centigrade) for one hour and in TrypLE for 10 minutes. Dissociated cells were plated on coverslips in 6-well dishes and allowed to set for overnight. Post 6Gy irradiation, cells were fixed with 3.0% paraformaldehyde for 20 minutes (room temperature). Fixed cells were permeabilized using 0.2% Triton X-100 in PBS for 10 minutes. Blocking was performed using 3% FBS in PBS containing 0.1% Triton X-100 for 1 hour. The coverslips were immunostained using γH2AX (Millipore clone JBW301, Ser139, Cat#05-636) primary antibody, followed by fluorescently conjugated secondary antibodies (Alexa fluor) (Invitrogen). Mounting and staining of the nuclei were performed using Vectashield (DAPI; Vector Laboratories). The number of foci from 70–100 cells were manually counted across multiple microscope fields. For each field, the average number of foci was determined per cell. The average of the fields was

plotted in mean$\pm$SD format.

Immunofluorescence studies to detect RAD51 foci were carried out using a rabbit monoclonal against RAD51 (Sigma, clone 1M8, Cat#ZRB1492). For formalin fixed paraffin embedded tissues, 5-micron paraffin sections were de-waxed and rehydrated following standard protocols. Antigen retrieval consisted of steaming for 30 minutes using Citrate pH 6.0 antigen unmasking solution (Vector labs, H-3300-250). Slides were washed and equilibrated in TBS-Tween buffer (Sigma) for 10 min. The primary antibody was applied at a dilution of 1:50 at 37°C for 60 min followed by incubation for 30 with a prediluted secondary anti-rabbit antibody (Leica, Cat#PV6119,). Immunocomplexes were visualized using the Tyramide 568 SuperBoost kit (Thermo Fisher, Cat# B40956) following manufacturer's instructions.

For *ex vivo* cultures and cell line experiments, cells were grown on coverslips and fixed for 10 min in 10% buffered formalin. After permeabilization (0.125% Triton-X in PBS) and blocking for 30 min in Antibody Dilution Buffer (ADB250, Ventana Indianapolis, IN) coverslips were incubated with primary antibody at 1:1000 dilution at 37°C for 60 min. Secondary antibodies and signal amplification were applied as described above. DNA was counterstained with DAPI (Sigma). Slides were visualized using a Nikon E800 fluorescence microscope (Nikon Instruments, Melville, NY). The number of cells with greater than 5 RAD51 foci were counted. For each experiment, three independent counts of 50 cells were performed per cell line and treatment arm.

Methylation analysis. Genome-scale methylation analyses were carried out using Infinium MethylationEPIC BeadChip arrays (Illumina). Among the 850K methylation sites, 32 probes covering *CHD1* gene body and 5'UTR including 12 probes covering putative promoter region of *CHD1* were identified and compared. Raw data were analyzed using the Minfi Bioconductor package. IGV track was used to manually curate CHD1 5' UTR for potential promoter hypermethylation (3, 4).

For *CHD1* locus-specific DNA methylation analyses a previously extensively validated assay combining methylated-DNA precipitation and methylation-sensitive restriction enzyme digestion

(COMPARE-MS) was used (5). In brief, DNA samples were digested with AluI and HhaI (New England Biolabs, Ipswich, MA) and methylated DNA fragments were enrichment using recombinant MBD2-MBD (Clontech) immobilized on magnetic Talon beads (Clontech, Mountain View, CA). Precipitated methylated DNA fragments were eluted and subjected to quantitative real-time PCR using the IQ SYBR Green Supermix (Biorad, Hercules, CA) with primers specific to the *CHD1* locus F: ACAGGTAAACGGATAGTGGGA, R: CTCGGTACTTTCTGGCTGGG. *In vitro* fully methylated male WBC DNA served as a positive control. For quantitative assessment of locus specific methylation levels, ct-values of the samples of interest were normalized to ct-values of the positive control and calculated methylation indices (ranging from 0.0 to 1.0) were used to derive methylation heatmaps.

DATA AVAILABILITY

The whole exome sequencing data sets generated during and/or analysed during the current study are available in the cBIOPortal (prad_su2c_2019) and Github under the accession: https://github.com/cBioPortal/datahub/tree/master/public/prad_su2c_2019. The ChIP-Seq data were downloaded from the Gene Expression Omnibus repository (GEO) under accession number GSE161948.

CODE AVAILABILITY

The code (R script) and a description of the approach for the iHRD classification is available through GitHub: https://github.com/ndesar/iHRD-Predict.R.

SUPPLEMENTAL REFERENCES

1. Mundra PA, and Rajapakse JC. SVM-RFE with MRMR filter for gene selection. *IEEE Trans Nanobioscience.* 2010;9(1):31-7.
2. Dasgupta S, Goldberg Y, and Kosorok MR. Feature Elimination in Kernel Machines in Moderately High Dimensions. *Ann Stat.* 2019;47(1):497-526.
3. Robinson JT, Thorvaldsdottir H, Winckler W, Guttman M, Lander ES, Getz G, et al. Integrative genomics viewer. *Nat Biotechnol.* 2011;29(1):24-6.

4. Aryee MJ, Jaffe AE, Corrada-Bravo H, Ladd-Acosta C, Feinberg AP, Hansen KD, et al. Minfi: a flexible and comprehensive Bioconductor package for the analysis of Infinium DNA methylation microarrays. *Bioinformatics.* 2014;30(10):1363-9.
5. Yegnasubramanian S, Lin X, Haffner MC, DeMarzo AM, and Nelson WG. Combination of methylated-DNA precipitation and methylation-sensitive restriction enzymes (COMPARE-MS) for the rapid, sensitive and quantitative detection of DNA methylation. *Nucleic Acids Res.* 2006;34(3):e19.

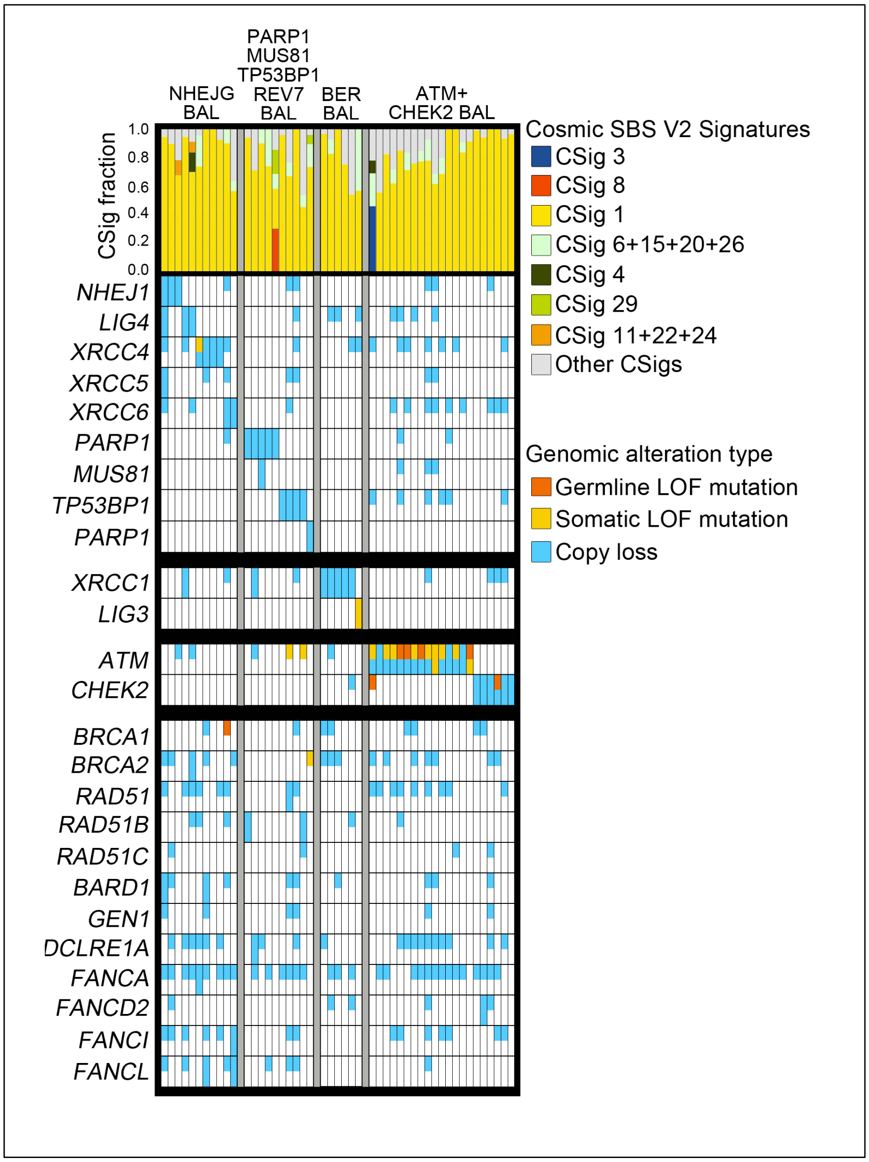

Supplementary Figure S1. COSMIC SBS mutation signatures in metastatic prostate cancers from the 418 mCRPC SU2C cohort with biallelic alterations in genes involved in NHEJ, non-homologous end-joining; BER, base-excision repair; and damage sensing.

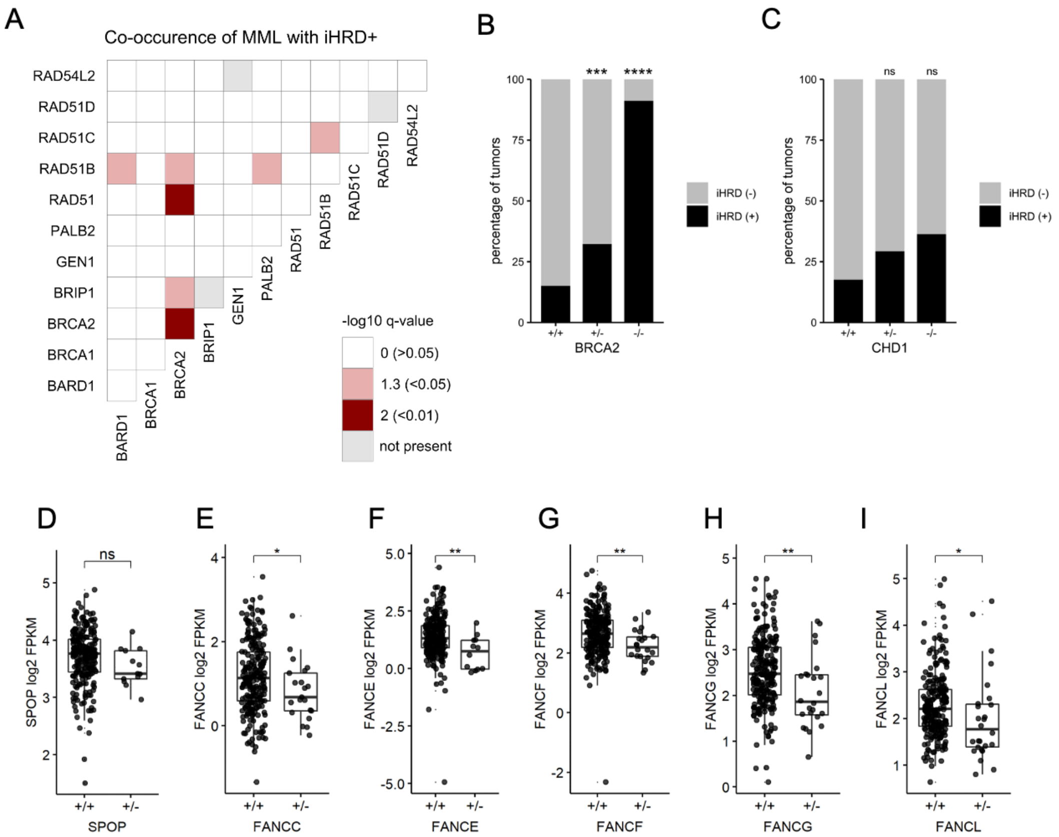

Supplementary Figure S2. Genomic alterations associate with the expression iHRD classification and the expression of HR repair genes. (A) Assessment of co-occurrence of mono-allelic aberrations in HRGs and tumors classified as iHRD(+). Fisher's exact tests of MML co-occurrence (or BAL for same gene pairs on the diagonal) versus iHRD status. Odds ratios were positive for all significant associations; p-value significance level color coded as red ($p<0.01$), pink ($p<0.05$), or white ($p>0.05$.) Grey boxes indicate gene pairs with no cooccurrence of MML or no BAL for same gene pairs. (B,C) Association of genomic loss of (B) BRCA2 or (C) CHD1 with iHRD status. Comparison of heterozygous (+/-) or homozygous (-/-) loss to wild type (+/+) by Fisher's exact test with Benjamini–Hochberg multiple testing correction shown on plot (FDR * ≤ 0.05; ** < 0.01; *** < 0.001; **** < 0.0001.) (D-I) Comparison of mRNA expression heterozygous (+/-) loss to wild type (+/+) by Wilcoxon-rank test with Benjamini–Hochberg multiple testing correction shown on plot (FDR* ≤ 0.05; ** < 0.01; *** < 0.001; **** < 0.0001.)

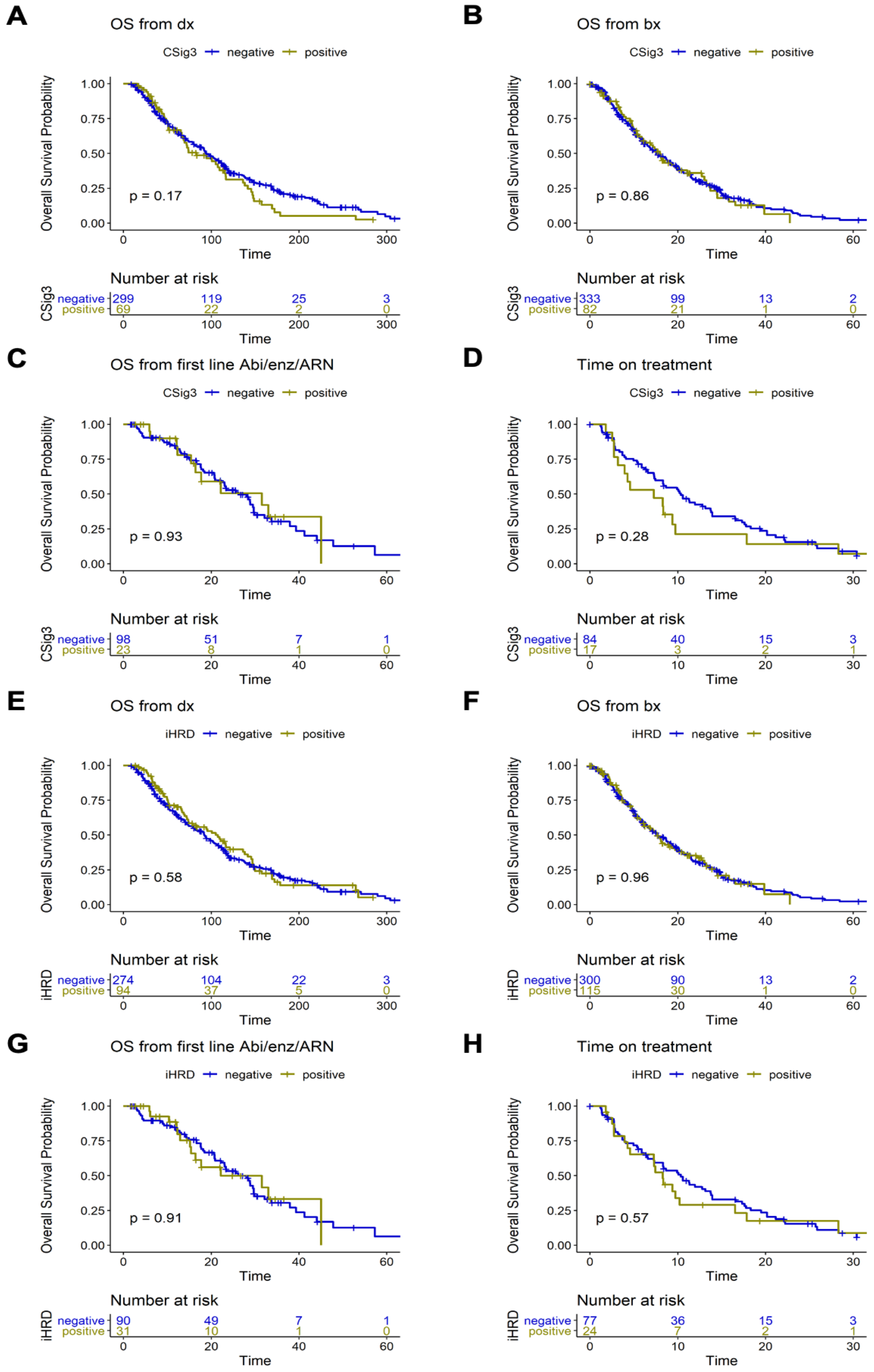

Supplementary Figure S3: Association of CSig3 and iHRD states with clinical outcomes. Kaplan–Meier analysis of (A,E) overall survival (OS) from the time of diagnosis, (B, F) and time of biopsy; (C, G) OS from initiation of first-line ARSi, and (D, H) time on ARSi therapy, grouped by CSig3 (A-D) and iHRD (F-H) status. Number of samples and log-rank test p-values are indicated on plots.

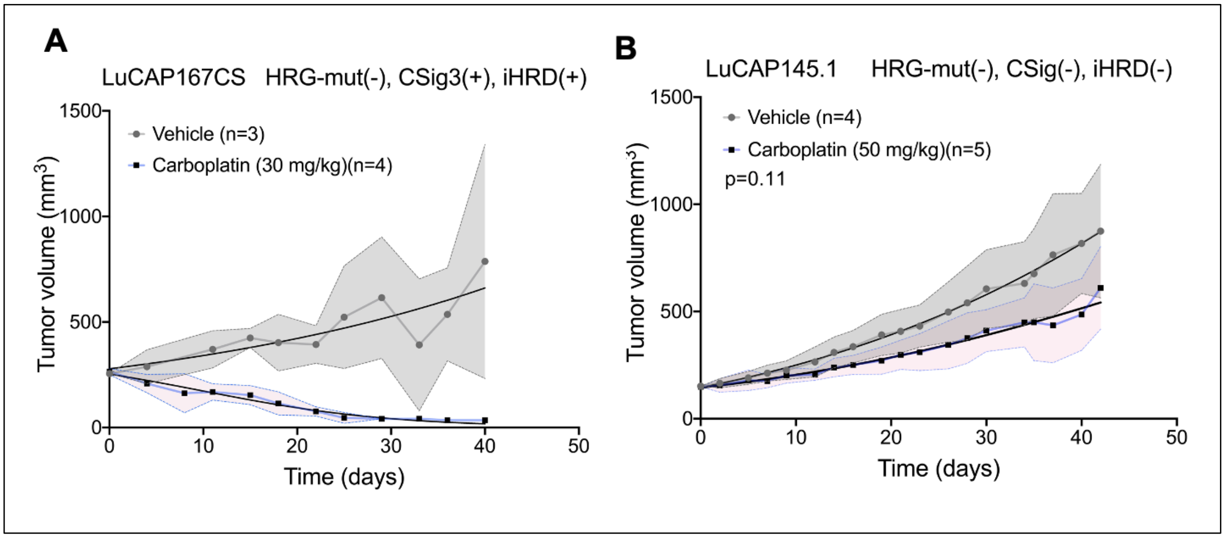

Supplementary Figure S4. Treatment outcomes associate with tumor CSig3 and iHRD classification. (A,B) LuCaP PDX tumor volumes measured over time in mice treated with carboplatin 30-50 mg/kg or vehicle control IP weekly (n=3-5 mice per group). Carboplatin significantly reduced the growth of iHRD+ LuCaP167 compared to vehicle ($p<0.01$). Carboplatin did not significantly impact the growth of iHRD(-) LuCaP 145.1 compared to vehicle treatment ($p=0.11$). In both plots, each time point is represented as mean tumor volume ± SD. Tumor growth curve comparisons were determined by permutation tests .

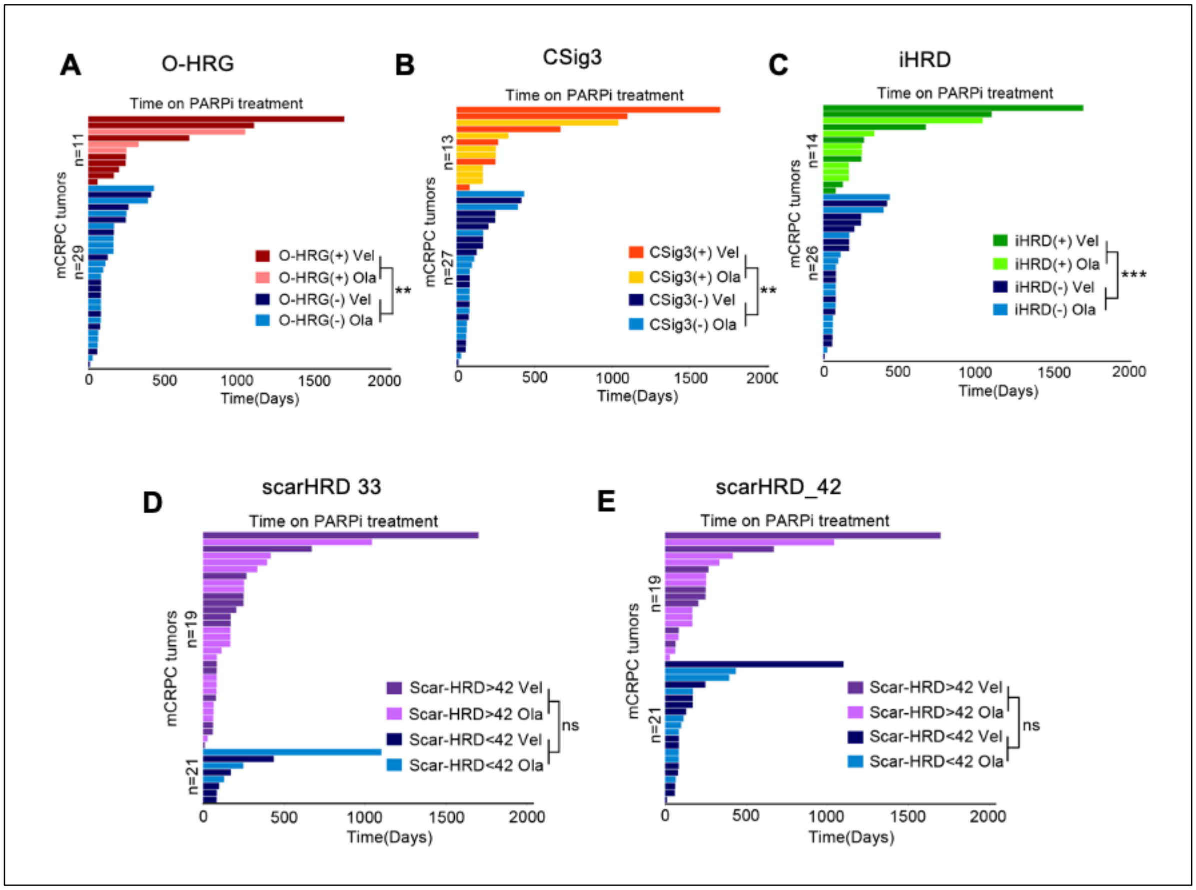

Supplementary Figure S5. PARPi treatment outcomes associate with tumor CSig3, iHRD and scarHRD classification. (A-E) Clinical outcomes for 40 patients treated with a PARP inhibitor shown by time (days) on drug depicted in swimmers plots partitioned by: (A) HRG mutation status: tumors are classified as O-HRG(+) or O-HRG(-) based on the biallelic loss of one of 14 genes approved for olaparib treatment; (B) CSig3 classification; (C) iHRD classification; (D) scarHRD with cutoff score 33, and (E) scarHRD with cutoff score 42. All comparison were made using the Mann-Whitney rank sum test. (* $p \leq 0.05$; ** $p < 0.01$, ns: nonsignificant).

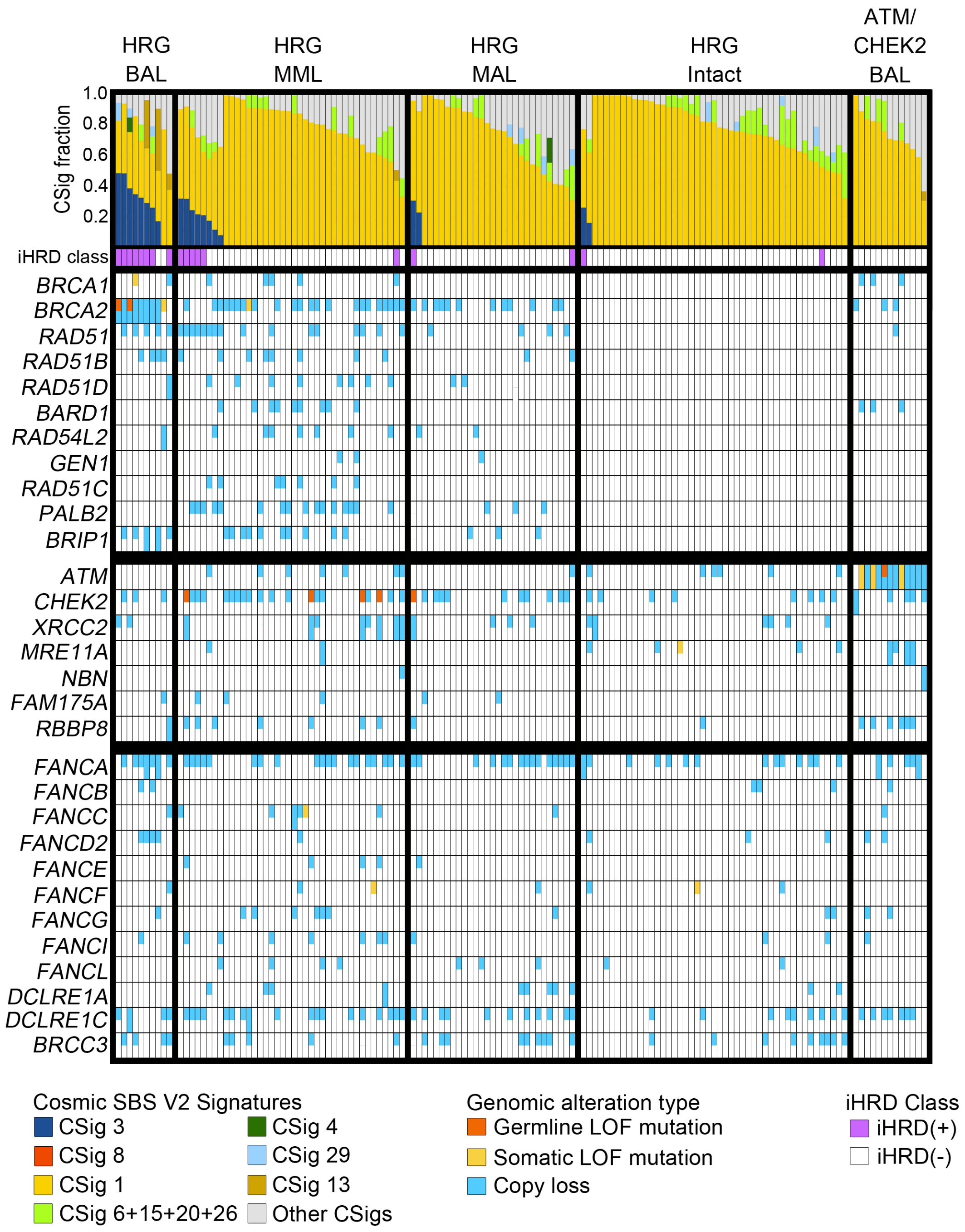

Supplementary Figure S6. University of Washington rapid autopsy cohort. Distribution of Cosmic single base substitution mutational signatures (CSigs) across metastatic prostate cancers resected at the time of rapid autopsy. Tumors are grouped by homology directed repair gene (HRG) germline mutation (HRG-Germ), HRG biallelic somatic loss (HRG-BAL), HRG multiple monoallelic loss (HRG-MML), HRG monoallelic loss (HRG-MAL), no HRG aberrations (HRG-Intact) and tumors with *ATM* or *CHEK2* biallelic loss mutation. The class of COSMIC mutation signatures are color coded and tumors are ordered in decreasing frequency of Cosmic signatures 3 (CSig3) fraction.

CHD1 antibody

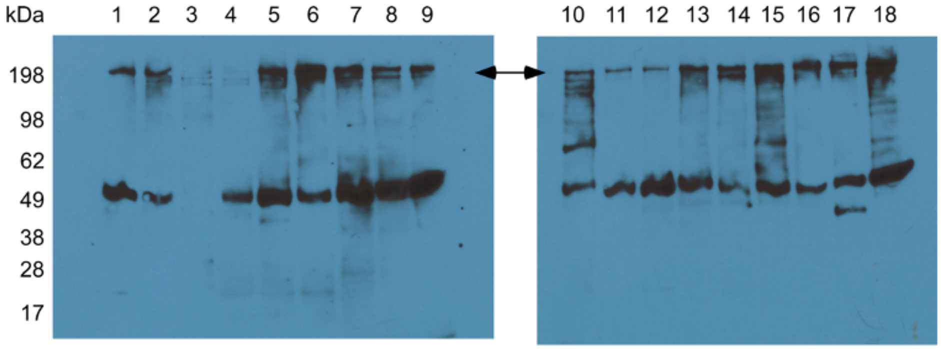

1. LUCAP 174.1
2. LUCAP 167CR
3. LUCAP 78CR
4. LUCAP 78
5. LUCAP70CR
6. LUCAP70
7. LUCAP173.1
8. LUCAP 189.3
9. LUCAP 189.4

10. LUCAP 145.2
11. LUCAP 92
12. LUCAP 170.3
13. LUCAP 170.1
14. LUCAP 170.2
15. LUCAP 147CR
16. LUCAP 147
17. LUCAP 173.2A
18. LUCAP 145.1

Western blot restained with GAPDH antibody

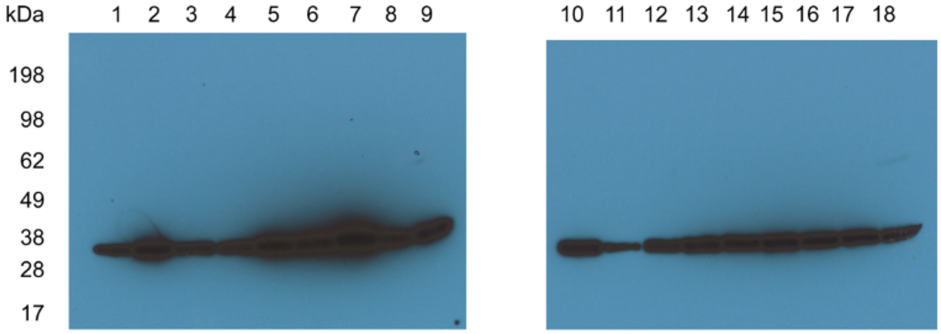

1. LUCAP 174.1
2. LUCAP 167CR
3. LUCAP 78CR
4. LUCAP 78
5. LUCAP70CR
6. LUCAP70
7. LUCAP173.1
8. LUCAP 189.3
9. LUCAP 189.4

10. LUCAP 145.2
11. LUCAP 92
12. LUCAP 170.3
13. LUCAP 170.1
14. LUCAP 170.2
15. LUCAP 147CR
16. LUCAP 147
17. LUCAP 173.2A
18. LUCAP 145.1

Supplementary Figure S7. Original CHD1 western blot shown in Figure 5 without crop. 10ug protein lysates from each of 18 PDX lines were loaded in each well.

CHD1 antibody

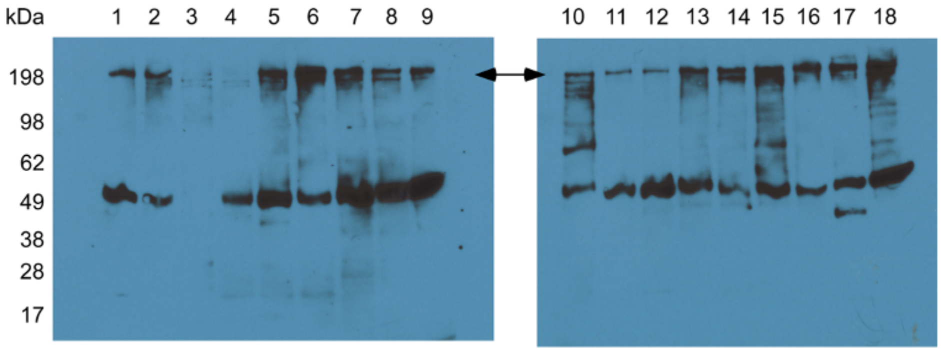

1. LUCAP 174.1
2. LUCAP 167CR
3. LUCAP 78CR
4. LUCAP 78
5. LUCAP70CR
6. LUCAP70
7. LUCAP173.1
8. LUCAP 189.3
9. LUCAP 189.4

10. LUCAP 145.2
11. LUCAP 92
12. LUCAP 170.3
13. LUCAP 170.1
14. LUCAP 170.2
15. LUCAP 147CR
16. LUCAP 147
17. LUCAP 173.2A
18. LUCAP 145.1

Western blot restained with GAPDH antibody

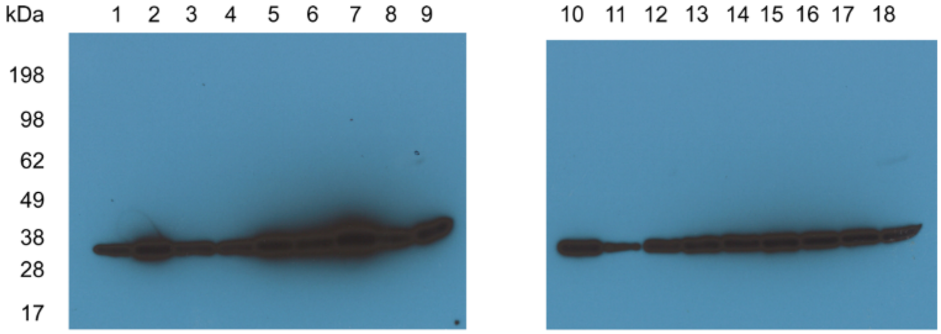

1. LUCAP 174.1
2. LUCAP 167CR
3. LUCAP 78CR
4. LUCAP 78
5. LUCAP70CR
6. LUCAP70
7. LUCAP173.1
8. LUCAP 189.3
9. LUCAP 189.4

10. LUCAP 145.2
11. LUCAP 92
12. LUCAP 170.3
13. LUCAP 170.1
14. LUCAP 170.2
15. LUCAP 147CR
16. LUCAP 147
17. LUCAP 173.2A
18. LUCAP 145.1

Uncut Immunoblot for Figure 5. Original CHD1 western blot shown in Figure 5 without crop. 10ug protein lysates from each of 18 PDX lines were loaded in each well.
